# Major limitations to achieving “4 per 1000” increases in soil organic carbon stock in temperate regions: Evidence from long‐term experiments at Rothamsted Research, United Kingdom

**DOI:** 10.1111/gcb.14066

**Published:** 2018-02-28

**Authors:** Paul Poulton, Johnny Johnston, Andy Macdonald, Rodger White, David Powlson

**Affiliations:** ^1^ Department of Sustainable Agriculture Sciences Rothamsted Research Harpenden UK; ^2^ Department of Computational and Analytical Sciences Rothamsted Research Harpenden UK

**Keywords:** 4 per 1000, carbon sequestration, climate change mitigation, long‐term experiments, management practices, organic amendments, Rothamsted, soil organic carbon

## Abstract

We evaluated the “4 per 1000” initiative for increasing soil organic carbon (SOC) by analysing rates of SOC increase in treatments in 16 long‐term experiments in southeast United Kingdom. The initiative sets a goal for SOC stock to increase by 4‰ per year in the 0–40 cm soil depth, continued over 20 years. Our experiments, on three soil types, provided 114 treatment comparisons over 7–157 years. Treatments included organic additions (incorporated by inversion ploughing), N fertilizers, introducing pasture leys into continuous arable systems, and converting arable land to woodland. In 65% of cases, SOC increases occurred at >7‰ per year in the 0–23 cm depth, approximately equivalent to 4‰ per year in the 0–40 cm depth. In the two longest running experiments (>150 years), annual farmyard manure (FYM) applications at 35 t fresh material per hectare (equivalent to approx. 3.2 t organic C/ha/year) gave SOC increases of 18‰ and 43‰ per year in the 23 cm depth during the first 20 years. Increases exceeding 7‰ per year continued for 40–60 years. In other experiments, with FYM applied at lower rates or not every year, there were increases of 3‰–8‰ per year over several decades. Other treatments gave increases between zero and 19‰ per year over various periods. We conclude that there are severe limitations to achieving the “4 per 1000” goal in practical agriculture over large areas. The reasons include (1) farmers not having the necessary resources (e.g. insufficient manure); (2) some, though not all, practices favouring SOC already widely adopted; (3) practices uneconomic for farmers—potentially overcome by changes in regulations or subsidies; (4) practices undesirable for global food security. We suggest it is more realistic to promote practices for increasing SOC based on improving soil quality and functioning as small increases can have disproportionately large beneficial impacts, though not necessarily translating into increased crop yield.

## 1 INTRODUCTION

The “4 per 1000 initiative: Soils for Food Security and Climate”, also known as “4 per mille” or “4‰”, for increasing soil organic carbon (SOC) stocks was launched by the French Ministry of Agriculture in 2015 in preparation for the Paris climate conference of the United Nations Framework Convention on Climate Change (UNFCCC). It is described in several web sites including:

1. https://www.4p1000.org/4-1000-initiative-few-words
2. http://newsroom.unfccc.int/lpaa/agriculture/join-the-41000-initiative-soils-for-food-security-and-climate

It is also described by Chabbi et al. (2017) and Minasny et al. (2017). The aim of the initiative is to promote land management practices leading to an increase in the stock (i.e. quantity as opposed to concentration) of SOC at the rate of 4‰ (0.4%) of the initial value per year for 20 years. It was originally suggested that, if this rate of C sequestration was achieved for all soils globally to a depth of 40 cm, the C removed from the atmosphere would equal annual CO_2_–C emissions from fossil fuels of 8.9 Gt and thus “halt the annual increase in CO_2_ in the atmosphere” (http://4p1000.org/understand). Later enunciations of the initiative have recognized that increases in SOC are only likely in soils that are being actively managed for agriculture (and possibly under managed forestry) and, even in these, such a rate of increase may not be achievable everywhere (Chabbi et al., 2017; Chambers, Lal, & Paustian, 2016; Lal, 2016; Minasny et al., 2017). It is suggested that C sequestration in agricultural soils alone, as opposed to all soils, might be limited to *c*. 1 Gt/year (Chabbi et al., 2017; Smith, 2016). There is also some uncertainty about the soil depth referred to in the initiative; in some publications, the 0–100 cm depth is mentioned in addition to 0–40 and 0–30 cm (Chabbi et al., 2017). Minasny et al. (2017) published a compilation of data from 20 regions of the world showing opportunities and limitations for achieving the 4‰ rate of SOC increase.

The initiative has been generally welcomed as laudable because any contribution to climate change mitigation is helpful and equally, or perhaps more importantly, any increase in SOC is virtually certain to improve the quality and functioning of many soils. This is especially relevant in the light of the reported widespread incidence of land degradation globally (UNCCD, 2017). However, there have been significant criticisms of the initiative by several authors (van Groeningen, van Kessel, Hungate, & Oenema, 2017; Baveye, Berthelin, Tessier, & Lemaire, 2018; de Vries, 2018; VandenBygaart, 2018; White, Davidson, Lam, & Chen, 2018), suggesting that there are many situations where the 4‰ rate of increase in SOC is not feasible for land managers in practical situations and that some of the experimental examples given by Minasny et al. (2017) are not representative of what is practically achievable in wide‐scale agriculture. There also appear to be differences of opinion as to whether the 4‰ goal is a specific target, an aspiration goal or even “more of a concept (or even a slogan)” as suggested by Minasny et al. (2018).

The aims of this paper were twofold. First, to report the rates of SOC increase in several long‐term agricultural field experiments run by Rothamsted Research at three sites in southeast England between 1843 and 2013. In temperate climates, SOC changes slowly in response to changes in management so periods of years or decades are required to reliably detect and quantify rates of change (Macdonald et al., 2015; Storkey et al., 2016). We use data from 16 separate experiments on three different soil types, giving over 110 treatment comparisons. It is recognized that the sources of data are geographically constrained but the cropping systems are diverse, and treatments cover a wide range of management practices relevant to temperate regions, though do not include reduced tillage. The results represent the largest concentration of long‐term SOC data globally, and the soil types and climates are broadly representative of temperate regions globally. Second, we attempt to use the long‐term data to provide an evidence‐based assessment of the likely range of situations where the 4‰ target might be achieved in practical agriculture, and for how long increases at this rate might continue.

## 2 MATERIALS AND METHODS

### 2.1 Experimental sites and soils

Soil samples from 16 experiments with a cool temperate climate in southeast United Kingdom were analysed for their organic carbon content. Much of the data have not been published previously and for several experiments, data have been updated. The soil types are silty clay loam at Rothamsted, sandy loam at Woburn and sandy clay loam at Saxmundham (see Table 1 for soil classifications). Long‐term average annual rainfall is *c*. 700, 650 and 610 mm at Rothamsted, Woburn and Saxmundham respectively. Until the late 1980s, the mean annual temperature was *c*. 9.0–9.4°C at the three sites, but, at Rothamsted and Woburn, where meteorological recording has continued, it has increased by about 1°C in the last 25–30 years (Scott, Macdonald, & Goulding, 2014; Johnston, Poulton, Coleman, Macdonald, & White, 2017). In all‐arable experiments, soils were cultivated by inversion ploughing to a depth of 20–22 cm.

**Table 1:** Experimental site details

Site, experiment and number	Duration	Clay (%)	Sampling depth (cm)	Crop	Treatments include	Reference
Rothamsted^a^, Hertfordshire, United Kingdom						
Broadbalk (1)	1843—ongoing	20–38	23	W. wheat	Rates of N, FYM, straw	Johnston and Garner (1969), Rothamsted Research (2006)
Hoosfield Barley (2)	1852—ongoing	18–27	”	S. barley	Rates of N, FYM, FYM residues	Jenkinson and Johnston (1977)
Fosters Ley‐arable (3)	1949—ongoing	”	”	Arable crops and leys	All‐arable rotations, 3‐year leys + 3‐year arable rotations, or permanent grass since 1949	Johnston (1973), Johnston et al. (2009)
Highfield Ley‐arable (4)	1949—ongoing	”	”	Arable crops and leys	All‐arable rotations, 3‐year leys + 3‐year arable rotations, or permanent grass since 1838	Johnston (1973), Johnston et al. (2009)
Amounts of Straw (5)	1986–2015	”	”	W. wheat	Wheat straw incorporated since autumn 1986	Powlson, Glendining, Coleman, and Whitmore (2011)
Broadbalk Wilderness (6)	1881—ongoing	20–25	”	Regenerating woodland	None	Poulton et al. (2003)
Geescroft Wilderness (7)	1886—ongoing	”	”	Regenerating woodland	None	Poulton et al. (2003)
Park Grass (8)	1856—ongoing	18–27	”	Permanent pasture	FYM	Warren and Johnston (1964), Fornara et al. (2011)
Exhaustion Land (9)	1852—ongoing	18–27	”	Arable crops^b^	FYM, FYM residues	Johnston and Poulton (1977), Johnston et al. (2017)
Woburn^c^, Bedfordshire, United Kingdom						
Amounts of Straw (10)	1986–2015	c. 14	”	W. wheat	Wheat straw incorporated since autumn 1986	Powlson, Glendining, et al. (2011)
Organic Manuring (11)	1965—ongoing	8–13	”	Arable crops and leys	Various organic amendments	Mattingly et al. (1974)
Green Manuring (12)	1936–67	c.13	20	Arable crops	Various organic amendments	Chater and Gasser (1970)
Market Garden (13)	1942—ongoing	10–12	23	Various crops	Various organic amendments from 1942 to 1967	Johnston (1975)
Ley‐arable (14)	1938—ongoing	11–16	25	Arable crops and leys	All‐arable rotations, 3‐ and 8‐year leys + 2‐year arable rotations, FYM every 5 years until mid‐1960s	Johnston et al. (2017)
Saxmundham^d^, Suffolk, United Kingdom						
Rotation II (15)	1899–1986	c. 25	20	Arable crops	FYM every 4 years	Mattingly, Johnston, and Chater (1969)
Rotation I (16)	1899–2009	”	”	Arable crops	FYM	Williams and Cooke (1971)

a The soil at Rothamsted is a flinty silty clay loam over Clay‐with‐flints and is classified as a Chromic Luvisol (IUSS Working Group WRB, 2015).

b Potatoes were grown continuously from 1876 to 1901; FYM was applied annually during this period.

c The soil at Woburn is a sandy loam and is classified as a Cambic Arenosol (IUSS Working Group WRB, 2015).

d The soil at Saxmundham is a sandy clay loam derived from boulder clay and is classified as a Eutric Gleysol (IUSS Working Group WRB, 2015).

Table 1 lists the experiments with references to publications giving details. In subsequent Tables, data are grouped according to treatment, taking data from different experiments where required rather than discussing each experiment separately. Further information appropriate to this paper is in Supporting Information.

### 2.2 Soil sampling and analysis

Most soils were sampled to 23 or 25 cm, i.e. a little deeper than current plough depth, with a semicylindrical gouge auger (2–3 cm diameter), taking many cores per plot. However, early samples on Broadbalk and Hoosfield were taken from several positions within each large plot with an open‐ended iron box sampler (typically 30.5 × 30.5 × 22.9 cm deep). Each “box” sample provided a large mass of soil which made it possible to determine the weight of fine soil (<6 mm, the standard procedure at Rothamsted at the time for samples that were to be archived) per unit volume. Similar box samples have been taken from many long‐term experiments at Rothamsted, Woburn and Saxmundham and the measured soil weight is used for these and other experiments on the same soil type. Soils were air‐dried and subsamples ground to <355 μm for nitrogen (N) and carbon (C) analysis. Organic carbon was determined by Tinsley (1950) or by modified Walkley‐Black (corrected to make the data equivalent to Tinsley; Kalembasa & Jenkinson, 1973). Later soils, and some earlier archived soils, were analysed for total C by combustion (LECO Corp., St Joseph, Michigan, USA) and a correction made for inorganic C, determined by manometry (Skalar Analytical BV, Breda, Netherlands). Standard soils are included with each batch of soils sent for analysis. For recent analyses, the mean and standard error of the standard soil is 1.46% OC ± 0.005.

### 2.3 Correcting amounts of SOC for changes in soil bulk density

When measuring changes in the quantity (stock) of organic C in the soil over time, it is important, where possible, to sample the same weight of mineral soil each time (Jenkinson, Poulton, & Bryant, 2008), on the assumption that most organic matter is held on the mineral particles. Where bulk density has not changed and, therefore, the weight of soil to a defined depth has not changed, the soil is sampled to the same depth. But, where bulk density has changed allowance must be made for this as discussed in detail in Johnston et al., 2017. In some of the experiments discussed here bulk density has declined due to addition of bulky organic material or a period in pasture, and later soil samples should have been taken to a slightly greater depth so that SOC is determined in the same weight of mineral soil. If this is not done, and the same weight of soil is used for the start and end of the measured period, this will overestimate the amount of C being sequestered. If the soil weight has been measured at the start and end of a period, but no allowance is made for the mass of extra soil which should have been sampled, and the amount of C it contained, then the amount of C being sequestered will be underestimated. Where bulk density has declined and where the weight of soil has been determined we have made a simple correction for that change. If, for example, the soil weight to a depth of 23 cm at the beginning and end of a period in ley‐arable cropping was 3,770 t/ha and 3,470 t/ha respectively, then the difference, 300 t/ha, represents the amount of “extra” soil that should have been sampled at the end (see Johnston et al., 2017). The amount of C in this extra soil can be calculated (it will have the same concentration of C as at the start) and added to that in the sampled layer. Where the amounts of SOC have been adjusted for bulk density changes, they are given in the appropriate Tables.

### 2.4 Relating our data for increases in SOC with the 4‰ target

The rates of increase in SOC calculated from our data are for soil samples taken to a depth of 20, 23 or 25 cm. Where possible, we have taken account of any extra soil that should have been sampled where bulk density declined over time as a result of the treatment (see above). However, the “4 per 1000” initiative refers to increases in SOC to a depth of 40 cm. In Supporting Information, we describe how we can adjust our data to that for 0–40 cm. Based on these calculations, we suggest that, for most of the topsoil data presented here, an increase of 7‰ equates to 4‰ when expressed on a 0–40 cm basis. Where the topsoil contains a higher concentration of OC (e.g. in grassland or woodland sites or where large amounts of manure have been applied) an increase of *c*. 5‰ will equate to 4‰ for the 0–40 cm layer.

### 2.5 Statistical analysis

All the experiments reported here are, or were, long‐term but for some we present changes over relatively short (e.g. 3–7 years), sometimes consecutive, periods (see Tables 5, 6, 9, S1–S5). Many of the experiments were established before the introduction of modern experimental design so that treatments were not necessarily replicated or randomized. Thus, the degree to which conventional statistics can be used to assess whether the observed changes are statistically significant is limited. Where errors have been calculated, and published, these are given in the appropriate tables. In other cases, where possible, the standard error of the mean of the differences between replicate plots for the stock of SOC at the start and end of each period is given together with the standard error of the annual rate of increase.

It is clear from these long‐term experiments that measuring SOC differences between treatments with sufficient precision to assign statistical significance is challenging due to soil spatial variability and the large background of SOC against which changes occur. Reliably detecting the relatively small changes likely to result from application of the “4 per 1000” initiative will be even more difficult for the reasons discussed by Smith (2004).

### 2.6 Fitting curves to the accumulation of soil organic carbon on farmyard manure‐treated plots on the Hoosfield Barley and Broadbalk Wheat experiments

It is well established that the accumulation of SOC does not increase indefinitely but moves towards an equilibrium value (Johnston, Poulton, & Coleman, 2009; Smith, 2014; Gollany et al., 2011) so the accumulation of SOC can be represented by a curve. It was possible to fit such curves to the accumulation of SOC in farmyard manure (FYM)‐treated plots in the Hoosfield Barley and Broadbalk Wheat experiments because the soils have been sampled sufficiently frequently. An advantage of this procedure was that it enabled data from the two or three FYM treatments in the experiments that were started at different times to be amalgamated, thus giving some degree of replication. Changes in SOC between each sampling point are given in Table S1, making allowance for the SOC in any soil to a greater depth that should have been sampled due to decreasing soil bulk density (see above). For both experiments the exact starting value has been estimated using the approach described by Jenkinson and Johnston (1977) because no samples were taken at the start of these two experiments. The FYM‐treated plots are not replicated but soil from the several large box samples (see above) taken periodically from each large plot have been analysed separately for SOC. For each experiment, a simple limiting exponential model was fitted (GenStat^®^, 2016) using the following equation:

$$\text{org C}=a+b\times r^{\mathrm{time}}$$

where org C is SOC, in t/ha, *a* is an asymptote representing the maximum capacity of the soil for SOC under the specific management, the slope coefficient, *b*, represents the available capacity of the soil to take up SOC and *r* is the exponential coefficient, representing the rate at which the soil accumulates SOC.

On Hoosfield, one plot has received FYM every year since 1852, another since 2001. Using the start of each treatment as year zero the accumulation of SOC stocks over time was modelled for the combined dataset.

Broadbalk is more complicated; three plots receive (or have received) FYM; one since autumn 1843, one since autumn 1884 and one between autumn 1967 and autumn 2000 only. However, FYM was not applied to the two plots that started in 1843 and 1884 when these plots were fallowed, usually 1 year in five between 1926 and 1967, to control weeds (Johnston & Garner, 1969). Thus, for these two plots there is a break in the annual application of FYM between soil sampling in 1914 and that in 1967. This has been overcome by considering five separate series of data; 1843–1914 and 1967–2000 for the FYM plot since 1843; 1885–1914 and 1967–2000 for the plot since 1884; and 1967–2000 for the plot receiving FYM since 1967 (Table S1). However, there is no reason to suppose that after annual applications of FYM resumed the sequestration of SOC would not continue as before. We therefore considered that the separate series of measurements should belong to a common model. To construct a common time frame for the separate series, the *b* parameter was allowed to vary and then the calendar time values for each series were adjusted accordingly, back to the first set (1843–1914), while the *a* and *r* parameters were estimated from all the data, assuming that these would be the same for each series. The simple limiting exponential model (GenStat^®^, 2016) was then fitted to the time‐shifted data.

Each fitted curve accounts for a large proportion of the variance, 94% and 89% for Hoosfield and Broadbalk respectively, suggesting that any inferences drawn from the data are reliable. From each curve the amount of SOC in the soil after e.g. 20, 40, 60 years was estimated, and the average rate of accumulation in each 20‐year period calculated assuming a linear increase in SOC during that period, though recognizing that this is an approximation. These values were then used in comparisons with data from other experiments. Associated standard errors were calculated using GenStat^®^ (2016), but they should be regarded as approximate.

## 3 RESULTS

### 3.1 Comparing our data with the 4‰ initiative

The “4 per 1000” initiative relates to SOC in the 0–40 cm depth of soil but data from the long‐term experiments described in this paper are mostly derived from sampling to a depth of 0–23 cm, slightly deeper than the usual plough depth for arable soils at these sites. In Supporting Information, we describe how 0–23 cm data can be approximately related to 0–40 cm. In general, for most topsoil data presented, an increase of 7‰ in the 0–23 cm layer is equivalent to 4‰ in 0–40 cm depth. Where the topsoil has a higher SOC concentration (>*c*. 1.3%) an increase of *c*. 5‰ will equate to 4‰ for 0–40 cm soil.

### 3.2 Effects of farmyard manure additions on SOC stocks

Applying animal manure to soil is probably the oldest and most thoroughly researched means of adding nutrients and, concurrently, increasing the concentration and stock of SOC. Ten experiments on three soil types provide data on the rate of SOC increase in Tables S1–S3, with one that includes FYM applications in conjunction with different crop rotations. In the oldest of the Rothamsted experiments in Table 1, Broadbalk (started autumn 1843, growing winter wheat); and Hoosfield (started 1852, spring barley), FYM has, with some exceptions (see Materials and Methods), been applied every year at 35 t fresh material per hectare, *ca*. 8 t dry matter per hectare. This amount was tested for experimental purposes to supply large amounts of nutrients, not because it would be recommended as a practical treatment: the significance of this is discussed later.

Figurs 1 and 2 show the accumulation of SOC resulting from FYM applications in the Hoosfield and Broadbalk experiments respectively, the curves being constructed by amalgamating data from treatments in which FYM applications started at three different times (Broadbalk) and at two different times (Hoosfield) as described in “Materials and Methods”. The data used are shown in detail in Table S1. From the fitted curves, the rates of SOC increase, expressed as ‰ per year compared to the initial contents, have been calculated for different periods (Table 2).

**Figure 1:**
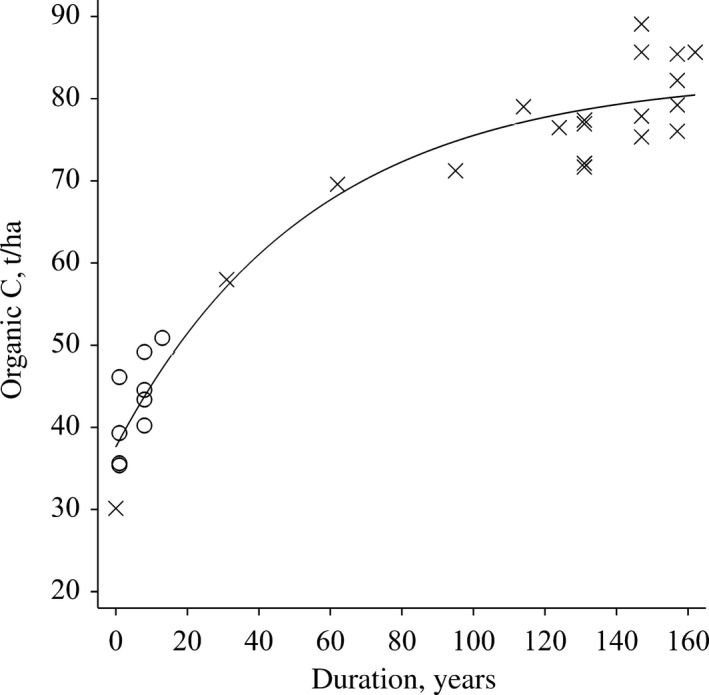
Accumulation of soil organic carbon (SOC), t/ha, resulting from an annual application of farmyard manure (FYM), 35 t/ha in the Hoosfield Barley Experiment, Rothamsted. The amount of SOC has been corrected for the change in soil bulk density at the end of each sampling period and an exponential curve fitted. FYM since 1852, (×); FYM since 2001, (○). The start of each treatment is considered as year zero

**Figure 2:**
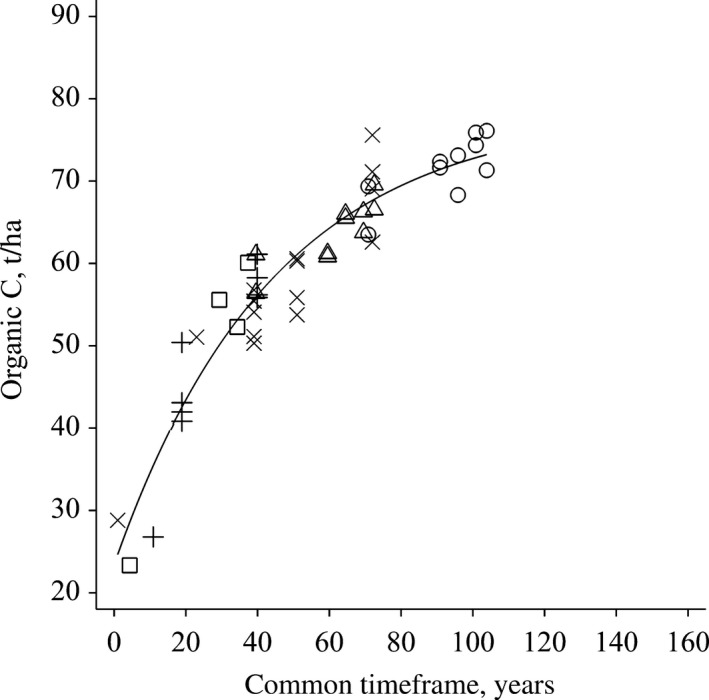
Accumulation of soil organic carbon (SOC), t/ha, resulting from an annual application of farmyard manure (FYM), 35 t/ha in the Broadbalk Wheat Experiment, Rothamsted. The amount of SOC has been corrected for the change in soil bulk density at the end of each sampling period. Each of the three series of data started in different years: Series 1. FYM from 1843 to 1914 (×), and again from 1967 to 2000, (○). Series 2. FYM from 1885 to 1914 (+), and again from 1967 to 2000, (∆). Series 3. FYM from 1967 to 2000 (□). To create a common timeframe, the calendar years for Series 2 and 3 data were shifted back to coincide with Series 1 before fitting the exponential model

**Table 2:** Predicted amounts of organic C in topsoil, together with standard errors, from exponential models, org C = *a *+ *b* × *r* ^time^, fitted to soils given 35 t FYM ha^−1^ year^−1^; Hoosfield^a^ and Broadbalk^b^

Experiment and number	Period (years)	Predicted amount of org C^c^		Rate of increase (t ha^−1^ year^−1^)	Annual increase (‰ /ha)
		At start (t/ha)	At end (t/ha)		
Hoosfield Spring barley (2)	1–20	37.6 ± 1.4	51.4 ± 2.0	0.69	8.4
	21–40	51.4 ± 2.0	61.0 ± 2.6	0.48	9.3
	41–60	61.0 ± 2.6	67.7 ± 2.5	0.34	5.5
	61–80	67.7 ± 2.5	72.3 ± 2.1	0.23	3.4
	81–100	72.3 ± 2.1	75.5 ± 1.5	0.16	2.2
	101–120	75.5 ± 1.5	77.7 ± 1.2	0.11	1.5
	121–140	77.7 ± 1.2	79.3 ± 1.2	0.08	1.0
	141–160	79.3 ± 1.2	80.4 ± 1.4	0.06	0.7
Broadbalk Winter wheat (1)	1–20	23.4 ± 3.5	43.4 ± 1.7	1.00	42.7
	21–40	43.4 ± 1.7	56.2 ± 1.2	0.64	14.7
	41–60	56.2 ± 1.2	64.3 ± 1.0	0.40	7.2
	61–80	64.3 ± 1.0	69.4 ± 1.4	0.26	4.0
	81–100	69.4 ± 1.4	72.7 ± 2.0	0.16	2.4
	101–120	72.7 ± 2.0	74.8 ± 2.6	0.10	1.4

a For Hoosfield: *a *=* *82.8 (*SE* 3.13); *b *=* *−45.2 (*SE* 2.89); *r *=* *0.982 (*SE* 0.005). Variance accounted for = 94%; *SE* of observations = 4.6.

b For Broadbalk: *a* = 78.5 (*SE* 4.50); *b* =* *−55.1 (*SE* 4.90); *r* =* *0.978 (*SE* 0.004). Variance accounted for = 89%; *SE* of observations = 4.0.

c Data to which the exponential models were fitted included any additional C present in the soil to an “equivalent” depth at the end of each period (see Supporting Information and Table S1).

Both experiments started with low stocks of SOC: 30.1 t/ha (1.15% C) in Hoosfield and 28.8 t/ha (1.00% C) in Broadbalk (Table S1). This is because the fields had been in arable cropping for several hundred years before the start of the experiments, probably in 5‐course rotations where FYM was applied 1 year in five. On Broadbalk it is known that no manure was applied in the 5 years prior to the start of the experiment in autumn 1843 (Lawes & Gilbert, 1885). In both experiments SOC initially increased rapidly with average rates of 18‰ and 43‰ per year in the first 20 years of applying 35 t/ha FYM annually in Hoosfield and Broadbalk, respectively. The rates remained above 7‰ per year (equivalent to 4‰ per year in 0–40 cm soil) for 40 years in Hoosfield and 60 years in Broadbalk (Table 2). After this, the rates declined as SOC stocks tended towards new equilibrium values and after about 80–100 years of applying this amount of FYM annually, the rate of change in SOC was not significantly different from zero.

The final SOC stock attained after applying FYM for many years was larger in Hoosfield (80 t C/ha) than in Broadbalk (75 t C/ha); see Figures 1 and 2. We can offer no complete explanation for this which may be due to small differences in soil properties or management between the experiments. In addition, on Broadbalk no crop was grown and no FYM applied 1 year in five for a 42‐year period when bare fallowing was used to control weeds (see Supporting Information and Table S1), so total organic C inputs over the course of the experiment were less than on Hoosfield.

In the Exhaustion Land experiment at Rothamsted, adjacent to Hoosfield and on the same soil type, FYM was applied from 1876 to 1901 at the same annual rates as on Broadbalk and Hoosfield. Soil samples taken in 1903 showed that the average rate of increase in SOC was 27‰ (Tables 3 and S2), a value comparable to the rates in Hoosfield and Broadbalk over similar periods.

**Table 3:** Changes in amount of organic C from adding farmyard manure

Experiment and number	Crop	Treatment^a^	Sampling period^b^	Amount of organic C^c^ in measured depth			Rate of increase (t C ha^−1^ year^−1^)	Annual increase (‰ /yr)	Notes
				At start (t/ha)	At end (t/ha)	Difference^d^ (t/ha)			
Saxmundham RII (16)	Various arable crops	FYM every 4th year	1899–1968	(43.9)	53.2	9.3 ± 1.25	0.13 ± 0.018	3.0	25.1 t/ha every 4th year
Woburn Green Manuring (12)	Various crops	FYM every 2nd year	1936–54	29.2	36.4	7.2	0.40	13.6	25.1 t/ha every 2nd year
Saxmundham RI (15)	Various arable crops	FYM every year	1899–1969	(36.5)	57.8	21.3 ± 1.75	0.30 ± 0.025	8.4	15.1 t ha^−1^ year^−1^ (30.1 t/ha since 1965)
Exhaustion Land (9)	Arable crops	FYM	1876–1903	(24.9)	43.8	18.9	0.68	27.1	35 t ha^−1^ year^−1^ 1876–1901
Woburn Organic Manuring (11)	Various arable crops	NPKMg	1965–1971^e^	25.8	24.6	−1.2 ± 0.37	−0.21 ± 0.062	−8.1	No organic amendment
	”	NPKMg	1980–1986	24.4	22.7	−1.7 ± 0.86	−0.28 ± 0.143	−12.0	”
	”	NPKMg	2002–2012	26.8	23.2	−3.5 ± 0.73	−0.35 ± 0.073	−13.2	”
	”	FYM	1965–1971^e^	25.2	35.3	10.1 ± 1.36	1.68 ± 0.227	66.4	50 t ha^−1^ year^−1^ + N applied 1965–1970
	”	FYM	1980–1986	29.4	36.8	7.4 ± 1.35	1.23 ± 0.225	42.2	50 t ha^−1^ year^−1^ + N applied 1981–1985
	”	FYM 10 t ha^−1^ year^−1^ since 2003	2002–2012	28.4	28.1	−0.3 ± 1.15	−0.03 ± 0.115	−0.2	10 t ha^−1^ year^−1^ + N
	”	FYM 25 t ha^−1^ year^−1^ since 2003	2002–2012	35. 4	40.0	4.6 ± 0.96	0.46 ± 0.096	13.1	25 t ha^−1^ year^−1^ + N
Park Grass (8)	Permanent pasture	FYM every 4th year since 1905	1923–1959	79.9	94.8	14.9 ± 5.54	0.41 ± 0.154	5.2	35 t ha^−1^ year^−1^ every
			1959–2002	94.8	104.2	9.4 ± 0.59	0.22 ± 0.014	2.4	4th year
	”	FYM every 4th year since 1905	1923–1959	77.9	98.0	20.1 ± 6.77	0.56 ± 0.188	7.4	35 t ha^−1^ year^−1^ every 4th year + NPK in other years
			1959–2002	98.0	97.4	−0.7 ± 1.61	−0.02 ± 0.037	−0.1	
	”	FYM every 4th year since 1905	1923–1959	87.6	85.1	−2.5	−0.07	−0.8	35 t ha^−1^ year^−1^ every 4th year + fishmeal (0.75 t/ha DM) every 4th year
			1959–2002	85.1	86.9	1.8 ± 2.87	0.04 ± 0.067	0.6	

a Unless stated, organic amendments were applied in autumn.

b Soils were sampled in autumn, before any organic amendments were applied; except Park Grass which was sampled in early spring. For Saxmundham RI and RII, starting values (in parentheses) are based on values from plots with NPK but no FYM input (RI) or no fertilizer or FYM (RII), measured in 1969 and 1968 respectively. For the Exhaustion Land the starting value (in parentheses) is the mean of data from control plots with no fertilizer or FYM input or from plots receiving NPK, measured in 1903.

c See Table S2 for soil weights and % OC determined by Walkley‐Black (corrected), Tinsley or combustion.

d ± *SE* of the mean of the differences between replicates where applicable.

e Sampled in spring 1964; there is assumed to have been no change between spring 1964 and autumn 1965.

Results from other experiments, at Saxmundham and Woburn (Table 3), better reflect FYM use in commercial practice, either because smaller amounts were applied annually and/or FYM was not applied every year. Although the treatments in these experiments were replicated, disadvantages are that the data are less detailed than in Broadbalk and Hoosfield as soils were sampled on fewer occasions and, in some cases, the treatments were applied for shorter periods. In the two experiments at Saxmundham, where the soil contains 25% clay, SOC increased significantly. The rates of increase were 3‰ per year averaged over 69 years where FYM was applied at 25.1 t/ha every 4 years and 8‰ per year averaged over 70 years where it was applied at 15.1 t/ha every year (Table 3). Thus, with an annual FYM application of 15.1 t/ha, the “4‰” goal (measured as 7‰ for the 0–23 cm soil depth) was exceeded over an extended period on this soil type, and it is likely that the rate of increase was much greater in the earlier years of each experiment.

In a much sandier soil (*c*. 13% clay) in the Woburn Green Manuring Experiment, starting at a lower SOC content, applying 25.1 t FYM/ha every 2 years increased SOC at an average rate of 14‰ per year, during a much shorter period of 18 years (Table 3). These results contrast with those in the Woburn Organic Manuring Experiment, also on a sandy soil and starting with only about 0.7% SOC. Treatments receiving only inorganic fertilizers lost SOC during different phases of the experiment. Large manure applications of 50 t/ha annually led to very rapid and significant increases of SOC (66‰ or 41‰ per year; Tables 3 and S2) when measured over shorter periods. In a later phase of the experiment, applying 25 t FYM ha^−1^ year^−1^ plus N fertilizer increased SOC significantly, at 13‰ per year over 10 years, but when only 10 t ha^−1^ year^−1^ was applied there was no increase. (Mattingly, Chater, & Poulton, 1974).

In the Park Grass experiment, started in 1856 on a site that had been in permanent pasture since *c*. 1700, applying FYM to the surface began in 1905 on three plots. FYM, at 35 t/ha, was applied every 4 years, either with or without additional inputs (see Supporting Information). Unfortunately, soil samples on these particular plots were not taken until 1923. Over the next 36 years there was a modest increase in SOC on two of the treatments and in the following 43 years there was little or no further change (Tables 3 and S2). In the Woburn Ley‐arable Experiment, started in 1938, different 5‐year crop rotations are compared, and initially in each rotation either no FYM or FYM applied once every 5 years at 38 t/ha was tested. In the rotations comprising 3 year of either lucerne or grazed grass followed by 2‐year arable, FYM increased SOC by 6‰ or 12‰ per year over 30 years (Table 4). Where only arable crops, mainly cereals, were grown, the rate of increase in SOC was smaller (3‰ per year) and where more root crops were grown in the 5‐year rotation there was no increase in SOC from applying FYM. Johnston et al. (2017) considered that these differences were due to the additional number of soil cultivations needed to grow root crops. Changes in SOC in the different rotations are described by Johnston et al. (2017) and summarized here in a later section.

**Table 4:** Changes in amount of organic C from adding farmyard manure every 5 years to continuous arable or ley‐arable rotations; Woburn Ley‐arable experiment (14)^a^

Crop rotation^b^	Treatment	No. of plots sampled	Sampling period	No. of years	Amount of organic C		Rate of increase (t C ha^−1^ year^−1^)	Annual increase (‰ /yr)	Notes
					At start (t/ha)	At end^c^ (t/ha)			
Ar	FYM every 5th year	5	1938–1965/74	30.5	37.0	37.0	0.00	0.0	213 t/ha applied from 1938 to the mid‐1960s
Ah	”	5	1938–1965/74	30.5	37.0	40.2	0.11	2.8	”
Lu3	”	5	1938–1965/74	30.5	37.0	43.4	0.21	5.7	”
L3	”	5	1938–1965/74	30.5	37.0	50.1	0.43	11.7	”

a Adapted from Tables 2, 4 and S2, Johnston et al., 2017. Although there was not a significant effect of FYM for individual rotations, when all rotations were considered together, there was a significant effect.

b Ar: 5‐year arable crops including root crops. Ah: 5‐year arable including 1‐year hay. Lu3: 3‐year lucerne ley followed by 2‐year arable. L3: 3‐year grazed grass/clover ley followed by 2‐year arable.

c For L3, the amount of organic C at the end includes additional C present in the soil to an “equivalent” depth; i.e. so that the same mass of mineral soil was being considered both at the start and end of the period.

### 3.3 Effects of various organic amendments on SOC stocks

Table 5 shows rates of SOC increase following annual applications of various organic amendments (the quantities added expressed as fresh weight) in two experiments on a sandy soil. The Woburn Market Garden Experiment was started in 1942 and data are available for a 25‐year period (Table S3). The soil contained only 0.87% C, equivalent to 31.1 t C/ha initially. Table 5 also shows the effect of adding compost for 10 years in the Woburn Organic Manuring Experiment where the initial SOC content was higher: 1.13% (Table S3), equivalent to 39 t C/ha.

**Table 5:** Changes in amount of organic C from adding various organic amendments

Experiment and number	Crop	Treatment^a^	Sampling period^b^	Amount of organic C^c^ in measured depth			Rate of increase (t C ha^−1^ year^−1^)	Annual increase (‰ /yr)	Notes
				At start (t/ha)	At end (t/ha)	Difference^d^ (t/ha)			
Woburn Organic Manuring (11)	Various arable crops	Compost	2002–2012	38.8	52.9	14.1 ± 2.42	1.41 ± 0.242	36.3	40 t ha^−1^ year^−1^ compost^e^
Woburn Market Garden (13) (Series A)	Market Garden crops	NPK	1942–1951	31.1	33.6	2.5	0.28	8.9	No organic amendment
			1951–1960	33.6	32.2	−1.4	−0.16	−4.7	”
			1960–1967	32.2	37.4	5.2 ± 0.87	0.82 ± 0.124	25.4	No organic amendment but amount of N applied doubled
	”	FYM	1942–1951	31.1	45.3	14.2	1.59	51.1	311.4 t/ha applied in total
			1951–1960	45.3	48.6	3.3	0.36	7.9	401.8 t/ha applied in total
			1960–1967	48.6	52.1	3.5 ± 1.51	0.51 ± 0.216	10.5	175.8 t/ha applied in total
	”	Vegetable compost	1942–1951	31.1	50.0	18.9	2.10	67.7	311.4 t/ha applied in total
			1951–1960	50.0	59.1	9.1	1.03	20.6	401.8 t/ha applied in total
	”		1960–1967	59.1	60.8	1.7 ± 2.49	0.20 ± 0.355	3.4	175.8 t/ha applied in total
	”	Sewage sludge	1942–1951	31.1	62.8	31.7	3.53	113.7	311.4 t/ha applied in total
			1951–1960	62.8	68.8	6.0	0.67	10.7	401.8 t/ha applied in total
	”	Sludge compost	1942–1951	31.1	53.6	22.5	2.50	80.5	311.4 t/ha applied in total
			1951–1960	53.6	63.2	9.6	1.07	20.0	401.8 t/ha applied in total
	”	FYM (double rate)	1942–1951	31.1	60.7	29.6	3.29	106.0	622.7 t/ha applied in total
			1951–1960	60.7	72.8	12.1	1.35	22.2	803.5 t/ha applied in total
	”		1960–1967	72.8	77.5	4.7 ± 4.3	0.66 ± 0.614	9.1	351.5 t/ha applied in total
	”	Vegatable compost (double rate)	1942–1951	31.1	60.3	29.2	3.25	104.7	622.7 t/ha applied in total
			1951–1960	60.3	71.4	11.1	1.23	20.4	803.5 t/ha applied in total
	”		1960–1967	71.4	80.2	8.8 ± 2.70	1.28 ± 0.385	17.9	351.5 t/ha applied in total
	”	Sewage sludge (double rate)	1942–1951	31.1	81.4	50.3	5.59	180.1	622.7 t/ha applied in total
			1951–1960	81.4	104.4	23.0	2.54	31.2	803.5 t/ha applied in total
	”	Sludge compost (double rate)	1942–1951	31.1	67.8	36.7	4.09	131.5	622.7 t/ha applied in total
			1951–1960	67.8	84.8	17.0	1.90	28.1	803.5 t/ha applied in total

a Unless stated organic amendments were applied in autumn.

b Unless stated, soils were sampled in autumn, before any organic amendments were applied.

c See Table S3 for soil weights and % OC determined by Walkley‐Black (corrected), Tinsley or combustion.

d ± *SE* of the differences between replicates where applicable.

e Commercially available compost supplying *c*. 24 t DM ha^−1^ year^−1^ and 300 kg N ha^−1^ year^−1^_._

In the Woburn Market Garden Experiment, four different organic materials (FYM and three different composts) were tested initially and all gave large annual rates of SOC increase in the first 9 years of 51‰–114‰ per year for annual applications of 35 t/ha annually of fresh material and even larger where a double rate was applied (Table 5). In the subsequent 9–18 years, SOC increases in excess of 7‰ per year continued (8‰–21‰ per year for the lower application rate). In 1960 two major changes were made to the experiment: only FYM and vegetable compost continued as organic amendments and the rate of N fertilizer applied annually to all treatments increased. During the third measurement period (18–25 years after the start of the experiment), SOC in the inorganic fertilizers (NPK) treatment increased at the rate of 25‰ per year, whereas in the FYM and vegetable compost treatments the rates of increase were smaller (11‰ and 3‰ per year). These smaller increases were presumably because the starting value of SOC in the organic amendment treatments in 1960 were considerably greater than in NPK (49 and 59 compared to 32 t C/ha; Table 5), and the SOC content was nearer its equilibrium value in the soils with organic amendments. The effect of N fertilizer on SOC stock is discussed later.

In the Woburn Organic Manuring Experiment, the rate of SOC increase in the first 10 years of compost application (at 40 t/ha annually) was slightly lower than in the Market Garden Experiment (36‰ per year), possibly because of the higher initial SOC content Table 5).

### 3.4 Effects of nitrogen fertilizer additions on SOC stocks

Increases in SOC following an increase in the annual mineral fertilizer N application are seen in the Broadbalk Wheat Experiment (Tables 6 and S4) although, due to lack of replication, we cannot ascertain whether these changes are statistically significant. In 1968, a plot that had received 48 kg N/ha since 1852 started to receive 192 kg N/ha, and during the next 20 years, SOC stock under continuous winter wheat increased at an average rate of 5‰ per year, and during the following 13 years it continued to increase at a similar average rate (Table 6). The N rate change from 1968 coincided with a change of crop cultivar to a short straw variety that gave considerably greater grain yields at all N rates, so the effects of the change in variety and increase in N rate cannot be unequivocally separated. However, total above‐ground dry matter production was almost equal between the new and old varieties at a given N rate (Austin, Ford, Morgan, & Yeoman, 1993) so it seems likely that organic C inputs to soil in roots and stubble were little changed due to the change in crop variety per se. It is therefore likely that the observed increase in SOC is associated with the increase in N fertilizer application. This could be caused by increased organic C input to soil resulting from increased crop growth or an increase in %N in crop residues caused by the increased N application facilitating increased C retention in soil, or a combination of these mechanisms.

**Table 6:** Changes in amount of organic C from increasing the amount of fertilizer N applied, adding straw or growing green manures or cover crops

Experiment and number	Crop	Treatment^a^	Sampling period^b^	Amount of organic C^c^ in measured depth			Rate of increase (t C ha^−1^ year^−1^)	Annual increase (‰ /yr)	Notes
				At start (t/ha)	At end (t/ha)	Difference^d^ (t/ha)			
Broadbalk (1)	W. wheat Sects 1 & 9	N applied increased from 48 to 192 kg/ha in 1968	1967–1987^e^	28.7	31.2	2.5	0.13	4.5	Also, a change from long to short straw cultivars in 1967
			1987–2000	31.2	33.4	2.2	0.17	5.3	
	”	N applied increased from 144 to 240 kg/ha in 1985	1987–2000	29.5	33.1	3.6	0.28	9.4	
	”	N applied increased from 96 to 288 kg/ha in 1985	1987–2000	30.1	35.7	5.6	0.43	14.4	
Broadbalk (1)	W. wheat Sect 0	Wheat straw incorporated since autumn 1986^f^	1988–2000	32.5	35.7	3.2 ± 1.30	0.26 ± 0.108	8.1	*c*. 36 t/ha straw applied in total
Woburn Green Manuring (12)	Various crops	Barley straw incorporated in alternate years	1936–1954	29.2	32.3	3.1	0.17	5.8	3.77 t/ha every 2 years
Woburn Organic Manuring (11)	Various arable crops	Wheat straw incorporated autumn 1965–1970	1965–1971^g^	28.5	32.5	4.0 ± 0.80	0.69 ± 0.133	24.1	45 t/ha straw applied in total
	”	Wheat straw incorporated autumn 1980–1985	1980–1986	28.2	29.6	1.4 ± 1.28	0.23 ± 0.213	8.1	45 t/ha straw applied in total
	”	Wheat straw incorporated since autumn 2002	2002–2012	31.4	31.5	0.1 ± 0.45	0.03 ± 0.045	1.1	75 t/ha straw applied in total
Woburn Green Manuring (12)	Various crops	Green manures	1936–1954	29.2	32.6	3.4	0.19	6.5	Spring sown lupins or rape or undersown clover or ryegrass
Woburn Organic Manuring (11)	Various arable crops	Green manures	1965–1971^g^	26.2	30.4	4.2 ± 1.62	0.75 ± 0.269	28.5	Ryegrass in 1964 and 1965; undersown trefoil in 1966 and red clover in 1968 and 1971
	”	Cover crops	2002–2012	30.3	24.8	−5.5 ± 1.06	−0.55 ± 0.106	−18.2	Overwinter cover crops in 2003/2004, 2006/2007, 2008/2009 and 2011/2012

a Unless stated, organic amendments were applied in autumn; fertilizer N was applied in spring.

b Unless stated, soils were sampled in autumn, before any organic amendments were applied.

c See Table S4 for soil weights and % OC determined by Walkley‐Black (corrected), Tinsley or combustion.

d ± *SE* of the differences between replicates where applicable.

e Sampled in autumn 1966; there is assumed to have been no change between autumn 1966 and autumn 1967.

f Mean of plots receiving 96, 144 and 192 kg N/ha + P, K and Mg.

g Sampled in spring 1964; there is assumed to have been no change between spring 1964 and autumn 1965. See Table 3 for effects on control treatment receiving NPKMg.

In 1985, two other treatments had their long‐term annual N application rates increased: one from 144 to 240 kg N/ha and one from 96 to 288 kg N/ha. The average rates of SOC increase measured over the next 13 years were 9‰ and 14‰ per year respectively.

As discussed in the previous section, increasing annual fertilizer N in the Woburn Market Garden Experiment, on a sandy soil, led to an average rate of increase in SOC stock of 25‰ per year over 9 years in the NPK treatment (Table 5). Although when expressed as ‰ per year this rate appears greater than in the Broadbalk examples, the absolute increase in the Woburn soil of 5.2 t C/ha is in the same range as in Broadbalk (2.2–5.6 t C/ha over the first 13–20 years; Table 6).

It is important to note that, in the situations described above, crop growth was not constrained by lack of other nutrients, inappropriate soil pH, or any other soil factors. Applying N fertilizer alone would not be expected to increase SOC if such conditions were not met.

### 3.5 Effects of straw incorporation, green manures or cover crops

Straw incorporation, at an average annual rate of 3 t/ha, was started in one section of the Broadbalk Experiment in 1987 on plots that initially had only 1.13% C. During the first 12 years, SOC increased significantly, at a rate of 8‰ per year, averaged over plots receiving 96–192 kg N/ha (Table 6).

In the Woburn Green Manuring Experiment, one treatment received straw at 3.77 t ha^−1^ year^−1^ every 2 years (Table 6). This increased SOC by an average of 6‰ per year over the next 18 years. In the Woburn Organic Manuring Experiment, straw was incorporated at different times and effects were compared with treatments receiving only inorganic fertilizers (shown in Table 3). In plots receiving only inorganic fertilizers, there were small decreases in SOC. Straw application led to variable increases ranging from zero to 24‰ per year measured over 6 or 10 years but, due to the variability, only the increase in SOC with largest rate of applied straw was statistically significant (Table 6).

In 1987, experiments comprising three rates of straw incorporation were started at both Rothamsted (18%–27% clay) and Woburn (14% clay). Where the amount of straw applied was equal to that produced, the average rates of SOC increase measured over 22 years were small and not significantly different from zero (Table 7). Where the rates of straw applied were two or four times the amount produced the increases were larger (5‰–10‰ per year) but statistically only the four‐times rate increased SOC significantly compared to no straw.

**Table 7:** Changes in organic C from adding various amounts of straw. Rothamsted and Woburn, Amounts of Straw experiments^a^

Site, experiment and number	Treatment^b^	Treatment period	Organic C^c^		Rate of increase^e^ (t C ha^−1^ year^−1^)	Annual increase (‰ /yr)	Notes
			At end (% C)	At end^d^ (t/ha)			
Rothamsted Amounts of straw (5)	No straw	1987–2008	1.86	53.2	—	—	No straw added
	Wheat straw incorporated since autumn 1986	1987–2008	1.96	56.1	0.13	2.4	100 t/ha straw applied
	Wheat straw × 2 incorporated since autumn 1986	1987–2008	2.07	59.2	0.27	5.1	201 t/ha straw applied
	Wheat straw × 4 incorporated since autumn 1986	1987–2008	2.14	61.2	0.36	6.8	402 t/ha straw applied
	SED		0.078 (differences in % C significant at *p* = .05)				
Woburn Amounts of straw (10)	No straw	1987–2008	1.00	34.4	—	—	No straw added
	Wheat straw incorporated since autumn 1986	1987–2008	1.10	37.8	0.16	4.5	87 t/ha straw applied
	Wheat straw × 2 incorporated since autumn 1986	1987–2008	1.21	41.6	0.33	9.5	175 t/ha straw applied
	Wheat straw × 4 incorporated since autumn 1986	1987–2008	1.22	42.0	0.34	10.0	340 t/ha straw applied
	SED		0.091 (differences in % C not significant)				

a Adapted from Table 2, Powlson, Glendining, et al. (2011).

b Straw was applied in autumn after soils were sampled.

c % OC determined by combustion.

d Using soil weights of 2,860 and 3,440 t/ha for Rothamsted and Woburn respectively.

e Compared to treatment without added straw.

Three experiments, all on sandy soil with an initial SOC concentration of <1%, provide limited sets of data on the impact of green manures or cover crops on SOC (Tables 6 and S2). In one case, green manures led to a small increase (7‰ per year over 18 years) and in another a combination of green manures and grass leys within a mainly arable rotation increased the rate to 29‰ per year, measured over 6 years and probably just statistically significant. In another treatment, inclusion of overwinter cover crops in 4 years out of 10 failed to increase SOC (Table 6).

### 3.6 Changing from continuous arable cropping to arable/ley rotations

The Woburn Ley‐arable experiment (Johnston et al., 2017), started in 1938, compares the effects of 3‐year arable cropping and 3‐year or 8‐year leys on the yields of two subsequent arable crops. Soils on which 3‐year lucerne leys were grown until 1970 showed only a very modest increase in SOC in the 0–23 cm soil layer over 30 years. This has been attributed to the wide‐spaced rows of lucerne with its main tap root rather than a dense mass of shallower roots, typical of grasslands (Johnston et al., 2017). When the lucerne ley was replaced by a grass/clover mixture, SOC increased by at least 4‰ per year over the next 35 years (Table 8). Where the 3‐year ley was grazed by sheep as opposed to being cut and the herbage removed, the rate of SOC increase was greater: 9‰ per year during 31 years (Table 8). But when the grazed grass/clover ley was changed to a grass ley given a small N fertilizer addition there was no further increase in SOC during the subsequent 35 years. Two cycles of 8‐year grass or grass/clover leys, followed by 2‐year arable cropping, were started in the 1970s. SOC increased at rates of 7‰–9‰ per year over the next 35 years (Table 8).

**Table 8:** Changes in amount of organic C in continuous arable or ley‐arable rotations. Woburn Ley‐arable experiment (14)^a^

Crop rotation^b^	Treatment	Sampling period	No. of years	Amount of organic C^c^		Rate of increase (t C ha^−1^ year^−1^)	Annual increase (‰ /yr)	Notes
				At start (t/ha)	At end^d^ (t/ha)			
Ar	Arable with root crops	1938–1965/74	30.5	36.95	35.25	−0.06	−1.5	Arable after long‐term arable
AF	Arable with 2‐year fallows	1965/74–2000/09	35	35.25	30.91	−0.12	−3.5	
Ah	Arable with 1‐year hay	1938–1965/74	30.5	36.95	38.64	0.06	1.5	”
AB	Arable	1965/74–2000/09	35	38.64	35.63	−0.09	−2.2	
Lu3	3‐year lucerne ley + 2‐year arable	1938–1965/74	30.5	36.95	40.34	0.11	3.0	Ley‐arable rotations after long‐term arable
LC3	3‐year grass/clover ley + 2‐year arable	1965/74–2000/09	35	40.34	46.24	0.17	4.2	
L3	3‐year grazed ley + 2‐year arable	1938–1965/74	30.5	36.95	47.18	0.34	9.1	”
LN3	3‐year grass ley with N + 2‐year arable	1965/74–2000/09	35	44.24	44.76	0.01	0.3	
LC8	8‐year grass/clover ley + 2‐year arable	1965/74–2000/09	35	41.28	51.69	0.30	7.2	”
LN8	8‐year grass ley with N + 2‐year arable	1965/74–2000/09	35	39.77	52.61	0.37	9.2	”
Rotation X year group *F*‐ratio_(10 302)_ = 35.40, *p* < 0.001								
SED for all amounts of organic C = 1.84								

a Adapted from Tables 2 and 4, Johnston et al. (2017). Data are the mean of plots without FYM and with FYM once every 5th year from 1938 to the mid‐1960s.

b Ar: 5‐year arable crops including root crops; becomes AF: 5‐year arable including 2‐year bare fallow. Ah: 5‐year arable including 1‐year hay; becomes AB: 5‐year arable. Lu3: 3‐year lucerne ley followed by 2‐year arable; becomes LC3: 3‐year grass/clover ley followed by 2‐year arable. L3: 3‐year grazed grass/clover ley followed by 2‐year arable; becomes LN3: 3‐year grass+N ley followed by 2‐year arable. LC8: 8‐year grass/clover ley followed by 2‐year arable. LN8: 8‐year grass+N ley followed by 2‐year arable.

c % OC determined by Tinsley or combustion.

d Includes any additional C present in the soil to an “equivalent” depth; i.e. so that the same mass of mineral soil was being considered at both the start and the end of each period.

Long leys (6 or 8 years) were also grown in the Woburn Organic Manuring experiment (Mattingly et al., 1974) where SOC increased significantly; increases ranged from 14‰ to 34‰ per year, measured at the end of the 6‐ or 8‐year period in the ley (Table 9). As noted for organic additions, SOC increases tended to be greater when the initial starting value was lower.

**Table 9:** Changes in organic C as a result of a change from long‐term arable or grassland to ley‐arable rotations, permanent pasture or continuous arable

Experiment and number	Crop	Treatment	Sampling period^a^	Amount of organic C^b^ in measured depth		Org C in extra soil^c^ (t/ha)	Total at end with extra organic C (t/ha)	Difference in organic C at start and end (t/ha)	Rate of increase (t C ha^−1^ year^−1^)	Annual increase (‰ /ha)	Notes
				At start (t/ha)	At end (t/ha)						
Woburn Organic Manuring (11)	Ley	8‐yr grass ley with N	1965–1971^d^	28.0	30.0	2.4	32.4	4.4 ± 0.96	0.75 ± 0.160	27.0	Ley after long‐term arable
		6‐yr grass/clover ley (previously grass ley with N)	1980–1986	29.8	29.8	2.6	32.6	2.6 ± 0.47	0.43 ± 0.078	14.5	Ley after 9‐year arable
		8‐yr grass/clover ley	1965–1971^d^	26.3	29.4	2.3	31.7	5.4 ± 0.53	0.9 ± 0.089	34.4	Ley after long‐term arable
		6‐yr grass/clover ley (previously grass/clover ley)	1980–1986	28.7	29.1	2.5	31.6	2.8 ± 0.60	0.47 ± 0.100	16.5	Ley after 9‐year arable
	Grass/clover	Grass/clover since 2003	2002–2012	31.3	35.0	2.7	37.7	6.4 ± 2.12	0.64 ± 0.212	20.6	Grass/clover after ley‐arable crops
Fosters Ley‐arable (3)	Arable cropping	NPKMg	1950–1987	41.0	37.8	0.0	37.8	−3.2	−0.09	−2.1	Arable after
			1987–2008	37.8	37.9	0.0	37.9	0.1	0.01	0.2	long‐term arable
	Ley‐arable cropping	3‐yr lucerne ley + 3‐yr arable rotation since 1949	1950–1987	41.0	42.4	0.0	42.4	1.4	0.04	0.9	Ley‐arable cropping after long‐term arable
		3‐yr grass ley with N + 3‐yr arable rotation since 1949	1950–1987	41.0	43.5	0.0	43.5	2.5	0.07	1.6	”
		3‐yr grazed grass ley + 3‐yr arable from 1949 to 1961/1962 then 3‐yr grass/clover ley + 3‐yr arable since 1962/63	1950–1987	41.0	43.7	0.0	43.7	2.7	0.07	1.8	”
			1987–2008	43.7	50.2	0.0	50.2	6.5	0.31	7.1	”
	Grass	Grass with N since 1949	1950–1987	41.0	60.2	2.1	62.3	21.3	0.57	14.0	Permanent grass with N after long‐term arable
			1987–2008	60.2	63.5	0.0	63.5	3.3	0.16	2.6	
Highfield Ley‐arable (4)	Arable cropping	NPKMg	1948–1987	61.3	39.6	−5.4	34.2	−27.1	−0.69	−11.3	Arable after grass since 1838
			1987–2008	39.6	37.9	−1.7	36.1	−3.5	−0.16	−4.2	
	Ley‐arable cropping	3‐year lucerne ley + 3‐year arable rotation since 1949	1948–1987	61.3	45.0	−8.4	36.6	−24.7	−0.63	−10.3	Ley‐arable cropping after grass since 1838
		3‐year grass ley with N + 3‐year arable rotation since 1949	1948–1987	61.3	45.9	−8.4	37.5	−23.8	−0.61	−9.9	”
		3‐year grazed grass ley + 3‐year arable from 1949 to 1961/1962 then 3‐year grass/clover ley + 3‐year arable since 1962/1963	1948–1987	61.3	45.7	−8.4	37.3	−24.0	−0.61	−10.0	”
			1987–2008	45.7	49.4	0.0	49.4	3.7	0.18	3.9	”
	Grass	Improved grass with N since 1949	1948–1987	61.3	66.5	0.0	66.5	5.2	0.13	2.2	Permanent grass with N after grass since 1838
			1987–2008	66.5	73.2	0.0	73.2	6.7	0.32	4.8	

a Unless stated, soils were sampled in autumn.

b See Table S5 for soil weights and % OC determined by Walkley‐Black (corrected), Tinsley or combustion.

c Where soil weight in the sampled depth has decreased, soils should have been sampled deeper so that the same mass of mineral soil was being considered. Where the soil weight has increased the soil should have been sampled less deeply; see text for further explanation.

d Sampled in spring 1964; there is assumed to have been no change between spring 1964 and autumn 1965. See Table 3 for effects on control treatment receiving NPKMg.

Ley‐arable experiments were started on two sites on silty clay loam soil at Rothamsted in 1949 (Johnston, 1973). One site, Fosters, had been in long‐term arable cropping and had an initial SOC content of about 1.5%. In this experiment, some plots were put into permanent grass, some into a rotation of 3‐year leys followed by 3‐years of arable crops and some plots continued to grow arable crops each year. Rotations that included a 3‐year grass + N ley or a 3‐year lucerne ley followed by 3 years of arable crops caused little increase in SOC compared to continuous arable (Table 9). Rotations that initially included grazed leys, but were replaced by cut grass/clover leys in the early 1960s, gave small increases in SOC (2‰–7‰ per year; Table 9). Soil subject to land use change from arable to permanent grass in 1949 gave an annual SOC increase of 14‰ per year over the following 37 years which then declined to 3‰ per year over the next 20 years (Table 9). The second Ley‐arable experiment at Rothamsted on Highfield was established on a site which had been in long‐term grass since 1838 (Lawes & Gilbert, 1885). The topsoil on this site contained about 2.75% SOC in 1949. All plots ploughed out of permanent grass lost SOC during the next 39 years whether in permanent arable or ley‐arable cropping (Table 9). Rotations which included 3‐year leys declined at a similar rate to soils in continuous arable rotations (Johnston et al., 2009).

### 3.7 Land use change from arable cropping to woodland

**Figure 3:**
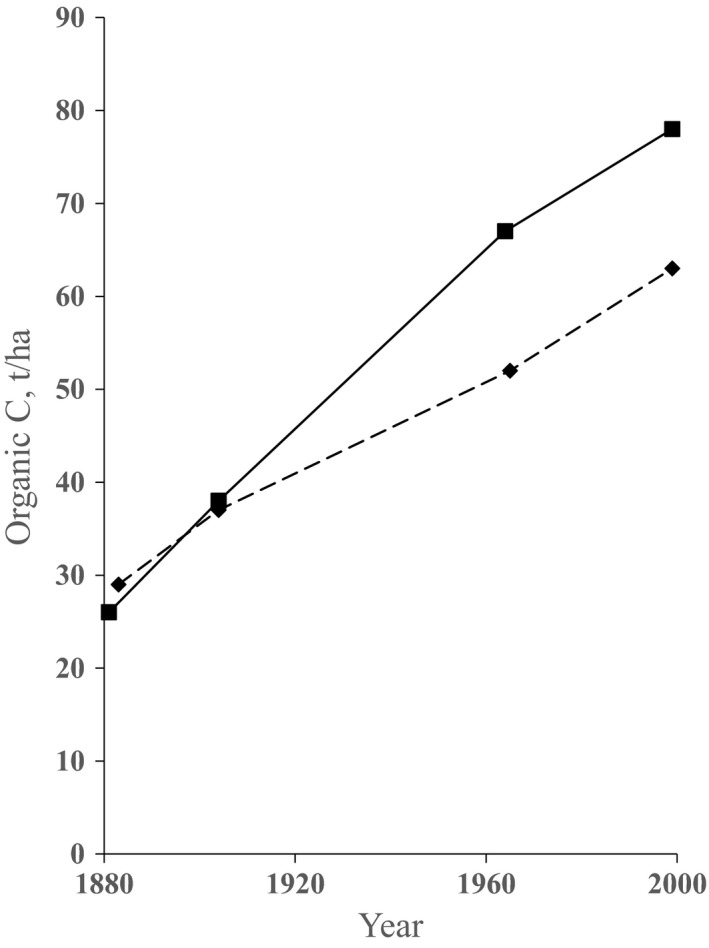
Accumulation of soil organic carbon (SOC), t/ha, in arable soils that were allowed to revert to woodland from the 1880s. The amount of SOC at the end of each sampling period has been corrected for the change in soil bulk density. Broadbalk Wilderness, (■); Geescroft Wilderness, (♦). Rothamsted

Figure 3 (summarizing data from Poulton, Pye, Hargreaves, & Jenkinson, 2003) shows SOC changes at two sites at Rothamsted following a major land use change from long‐term arable cropping to woodland through natural reversion. At one site, Broadbalk Wilderness, the soil initially contained calcium carbonate and remained above pH 7 for the entire period of over 110 years. In total this site accumulated 56% more SOC than the other site, Geescroft Wilderness, in which soil pH declined from 7.1 at the start of reversion in 1883 to 4.4 in 1999. During the first 20 years after reversion to natural vegetation, Geescroft and Broadbalk accumulated SOC at 15‰ and 19‰ per year respectively, declining to 10‰ and 5‰ per year in the final 30‐year measurement period. Besides the increase in SOC, much carbon was also accumulated in the above‐ground vegetation (Poulton et al., 2003). On these two woodland sites SOC had not reached a new equilibrium, even after >100 years (Figure 3). By contrast, when there was a change from old arable land to permanent grassland, SOC had reached an equilibrium after about 100 years (Johnston et al., 2009).

## 4 DISCUSSION

### 4.1 General considerations

Any increases in SOC will almost certainly improve soil functioning and quality. There is considerable evidence that even small increases can have disproportionately large and beneficial effects on soil biological activities and physical properties such as water infiltration, aggregate stability and ease of tillage (Haynes, Swift, & Stephen, 1991; Snyder & Vazquez, 2005; Blair, Faulkner, Till, & Poulton, 2006; Thierfelder & Wall, 2012; Thierfelder, Cheesman, & Rusinamhodzi, 2013; Verhulst et al., 2010; Watts, Clark, Poulton, Powlson, & Whitmore, 2006). For example, even where zero tillage increases SOC *concentration* in near‐surface soil (e.g. 0–10 cm), but with minimal impact on SOC *stock* to a greater depth, various soil physical properties can be considerably improved (Powlson, Stirling, Thierfelder, White, & Jat, 2016; Thierfelder & Wall, 2012). As pointed out by Janzen (2015), it is the process of decomposition of organic matter entering soil that delivers improvements in soil structure and functioning and the release of nutrients to crops: it is not necessary that a large increase in SOC stock is attained.

Where large amounts of FYM have been applied over many years much of the FYM‐C has been lost even though the increase in SOC has been large. For example, in the silty clay loam soil on Broadbalk only 11% of the 480 t organic carbon added in FYM since 1843 has been retained in the soil to a depth of 46cm (Table 10), yet the soil properties are drastically altered compared to that with no manure addition. In the treatment where FYM had only been applied since 1968, SOC was still far from reaching a new equilibrium level and a much larger proportion (35%) of the added C could be found. In the sandy loam at Woburn, modelling with RothC‐26.3, showed that 5%, at most, of the C added in plant residues over a 70‐year period is still present in the soil (Johnston et al., 2017) but, again, this caused measurable changes in soil properties. However, improvements in soil physical properties caused by increased SOC do not necessarily translate into consistently increased crop yields (Hijbeek et al., 2017).

**Table 10:** The retention of FYM‐derived C in the soil

Experiment and number	Treatment	Years applied	Soil sampled	FYM‐C applied (t/ha)	Additional C in soil compared to controls^a^		FYM‐derived C recovered compared to controls^a^	
					In topsoil 0–23 cm (t/ha)	In subsoil 23–46 cm (t/ha)	In topsoil 0–23 cm (%)	In subsoil 23–46 cm (%)
Broadbalk (1)	FYM 1843	Since 1843^b^	2000	477	39.8	13.2	8	3
	FYM 1885	Since 1885^b^	2000	341	33.8	12.5	10	4
	FYM 1968	1968–2000	2000	106	28.1	8.7	27	8
Hoosfield (2)	FYM 1852	Since 1852	1998	470	55.8	17.7	12	4
	FYM residues	1852–71	1998	64	10.5	0.6	16	1
Exhaustion Land (9)	FYM	1876–1901	1903	83	18.9	**—**	23	—
	FYM residues	1876–1901	1974	83	6.7	—	8	—

a For Broadbalk and Hoosfield, control treatments received N, P, K, Mg fertilizers; for the Exhaustion Land the control is the mean of plots receiving either no fertilizers or N, P, K, Mg fertilizers.

b Between 1926 and 1967, sections of the experiment were fallowed each year to control weeds. The two sections in continuous wheat being considered here (sampled in 2000) were fallowed nine times in this period (eight times on section 9; 10 times on Section 1); FYM was not applied in these years.

In contrast to the impacts of increasing SOC on soil properties and quality, the requirements for SOC increases for mitigating climate change through soil carbon sequestration are more stringent. First, it is necessary that SOC *stock* (i.e. the quantity of organic C in soil) is increased, not just the *concentration* of C in the surface layer. Second, it is essential that the additional C sequestered in soil would otherwise have been in atmospheric CO_2_ and is not simply being transferred from one terrestrial location to another. Other well‐known caveats must also be observed including the fact that the rate of SOC increase slows as the new equilibrium value is approached (Johnston et al., 2009; Powlson et al., 2012; Smith, 2014) and that increases are reversed if the modified management practice is not continued indefinitely (Powlson, Glendining, et al. 2011; Powlson, Whitmore, & Goulding, 2011; Mackey et al., 2013).

In considering the climate change mitigation potential of any change in land management practice, it is the overall impact on all greenhouse gas fluxes that must be assessed, not only changes in SOC (Smith et al., 2008). Some practices leading to C sequestration may increase emissions of trace greenhouse gases, especially N_2_O. For example, a recent global meta‐analysis of experiments with manure showed that, on average, manure addition increased N_2_O emission by 33% compared to inorganic N fertilizer and this could largely offset the benefit of increased SOC stock (Zhou et al., 2017). However, the trend was smaller with FYM than with manures containing a larger proportion of readily mineralizable N such as poultry manure.

Of the 114 treatment comparisons within the long‐term experiments reported in this paper, almost two‐thirds showed SOC stock increases >7‰ per year (or 5‰ in soils with larger initial SOC content); these increases, mostly measured in the 0–23 cm soil layer, being roughly equivalent to 4‰ goal specified for the 0–40 cm layer in the “4 per 1000” initiative. The increases were predominantly from organic inputs (manure, compost or straw) or from inclusion of pasture leys instead of continuous arable cropping. However, in evaluating the practicality of the “4 per 1000” initiative, considerable caution is required in transferring results from experiments such as these, and those reported by Minasny et al. (2017), to real world situations. Experimental results need to be evaluated in the light of the following practical and logistical considerations:

1. Is the practice suitable for a wide range of soil types and environmental conditions and possible for farmers to adopt in practical situations?
2. Is the practice profitable for a farmer? If the answer is “not under current conditions” but the practice is highly beneficial, either for climate change mitigation or long‐term soil quality improvement or food security, there could be an argument for changes in policy or financial arrangements to promote uptake of the practice.
3. What are the implications for global food security if a practice is widely adopted?
4. Is the practice already widely used in the regions concerned, thus giving limited opportunities for further adoption?

Below, we address the land management practices considered in this paper from the viewpoint of these considerations.

### 4.2 Removal of land from agriculture

Removing land from arable cropping, and allowing natural regeneration to deciduous woodland, led to large accumulations of organic C in soil in addition to that in trees; SOC stock doubled or trebled in a little over a century (Figure 3 and Poulton et al., 2003). Initially the rates of increase in these woodland reversion sites were very large and still exceeded 4‰ per year (in the 0–23 cm soil layer) during the final 30 years of measurement. Similarly, conversion from arable to permanent grass caused an increase of 55% in SOC in 58 years (Table 9). Piñeiro, Jobbágy, Baker, Murray, and Jackson (2009) also reported substantial increases in SOC at 142 sites in the United States where agricultural land was set aside. There are obviously severe limitations to the area of land that can be removed from agriculture globally if food security goals are to be met. But, in limited situations where soils are either of low productivity or are fragile and prone to erosion, conversion from agriculture to forest or grassland may be a logical strategy (Albanito et al., 2016; Smith et al., 2013). A good example is the “grain for green” programme in China that reduced soil erosion and led to considerable increases in SOC (Chadwick et al., 2015; Song, Peng, Zhou, Jiang, & Wang, 2014).

### 4.3 Addition of manures and other organic materials

The annual application of FYM at 35 t fresh material per hectare on Broadbalk since 1843 and Hoosfield since 1852 led to a high rate of SOC increase for several decades (Figures 1 and 2; Table 2) but such increases are unlikely to be achieved in practical farming situations. Few farmers would have such large quantities of animal manure available each year for all fields on their farm and, even if they did, they may be prevented from applying such amounts continuously because of the risk of nitrate and phosphate pollution (Goulding, Poulton, Webster, & Howe, 2000; Hesketh & Brookes, 2000) and/or government legislation (e.g. http://www.gov.uk/guidance/nutrient-management-nitrate-vulnerable-zones). However, on the positive side, even the more practically relevant application regimes (lower rates and/or applied every second or fourth year) in the Saxmundham and Woburn experiments led to rates of increase in SOC of 8‰–14‰ per year even when averaged over 20–70 years (Table 3). Thus, manure applications are a very effective way of increasing SOC, especially if used on soils with low initial SOC content where the potential for increase is greatest; the reduced rate of increase as SOC stock increased with time was very clear in all these experiments. This has important implications for farming practice: from the viewpoint of SOC management (and recycling of nutrients) it would be desirable to have a mixed landscape comprising farms with grass and arable fields in close proximity, as was the case some decades ago in northern Europe. Such mixed farming facilitates the application of manures to arable fields which tend to have a lower SOC content, and so benefit the most from organic additions, but is contrary to the current tendency in many countries for specialization, with animal and arable enterprises being spatially separated. Specialization is favoured by a range of practical (soil type and climate) and economic factors. If this trend is to be reversed to achieve environmental benefits, including increased SOC stocks, it is likely that significant changes in policies and financial incentives will be required. For example, if policies facilitated alterations in farm structures such that the estimated 300 Mt of solid manure produced in the European Union (Foged et al., 2011) were distributed more evenly this would be beneficial for soil quality.

While manure applications are very effective at increasing SOC and improving a wide range of soil functions, it should be recognized that these increases will generally not be delivering climate change mitigation but are rather a transfer of C from one location to another (Schlesinger, 2000). Globally, virtually all manure is currently being applied to soil at some location, though often in a suboptimal way. So, while there is certainly scope to make more rational and efficient use of manures both for soil C enhancement and for nutrient supply, almost all manure is already being used to some extent. Thus, it is incorrect to assume that all SOC increases observed in experiments on manure application can be transferred to practical situations and fully treated as climate change mitigation: at least part of the benefit will already be accruing (Powlson et al., 2012; Powlson, Glendining, et al., 2011; Powlson, Whitmore, et al., 2011). Where manure is being used inefficiently, or is applied to soil with an already high SOC stock, there is an opportunity to manage it differently by applying instead to low‐SOC soils with potential for some degree of climate change mitigation plus numerous other benefits for soil quality, nutrient supply and decreased water pollution (Chadwick et al., 2015). Although, of course, there are major practical barriers to transporting manure, even over moderate distances.

The different organic amendments tested in experiments on a sandy loam soil (sewage sludge, now commonly called biosolids, and various composts) all led to high rates of SOC accumulation (Table 5). As with most of the FYM treatments, the application rates were large so the absolute rates of SOC increase cannot be directly transferred to practical farming but there is a strong indication that, for a given application rate, they deliver larger increases in SOC than FYM and the effect is longer lasting. This is presumably because these materials have already undergone greater decomposition than FYM during composting or sewage treatment so the organic C applied to soil will be somewhat more recalcitrant (Johnston, 1975). An important factor regarding these materials is that they represent organic resources that are not currently widely utilized. In many countries, a significant proportion of food waste and similar organic materials is currently disposed of in landfill where decomposition returns CO_2_ or CH_4_ to the atmosphere (Bijaya, Barrington, & Martinez, 2006). Their greater use as a soil amendment can contribute to genuine climate change mitigation in addition to soil improvement (Powlson et al., 2012). However, it is recognized that some of these materials may have alternative uses that mitigate climate change in other ways, such as feedstock for anaerobic digestion to generate biogas as a substitute for fossil fuel. For example, about 8% of manure in the EU is currently processed for such purposes (Foged et al., 2011). To some extent these two uses can be combined if the digestate residue is applied to soil as a source of C and crop nutrients. Similarly, some organic wastes such as poultry litter are currently incinerated to generate electricity or heat, the residue being used as source of crop nutrients.

### 4.4 Retention of crop residues

Straw additions had variable, though generally positive, impacts on SOC stocks (Tables 6 and 7). Irrespective of soil type (silty clay loam at Rothamsted or sandy loam at Woburn), any increases tended to be greater where there was less SOC at the start of the experiment. Elsewhere, in temperate climatic regions, straw addition has also given positive but generally small increases in total SOC stock (Powlson et al., 2012; Powlson, Glendining, et al., 2011; Powlson, Whitmore, et al., 2011; van Groenigen et al., 2011). For example, in the 25 straw incorporation experiments of 6‐ to 56‐year duration reviewed by Powlson, Glendining, et al. (2011) and Powlson, Whitmore, et al. (2011), the increase in SOC was only statistically significant in six cases. However, as mentioned earlier, there is evidence that even small increases in SOC can have disproportionately large and beneficial effects on soil physical and biological properties (Houot, Molina, Chaussod, & Clapp, 1989; Malhi & Lemke, 2007; Ketcheson & Beauchamp, 1978; Thierfelder & Wall, 2012). In many regions, much cereal straw is already returned to soil (e.g. an estimate of 50% in the United Kingdom in 2008; Copeland & Turley, 2008) with much of the remainder being used for animal bedding and eventually returned to soil as FYM, so the scope for additional straw return to soil for climate change mitigation is limited. In smallholder agriculture in tropical regions the use of crop residues for animal feed or bedding is regarded as a significant barrier to direct return of crop residues to soil as part of wider adoption of conservation agriculture (Powlson et al., 2016; Thierfelder et al., 2013; Giller et al., 2011).

### 4.5 Conversion from continuous arable to ley‐arable cropping

In the experiments considered here this change of management often led to increases in SOC exceeding 7‰ per year (equivalent to 4‰ per year in the 0–40 cm depth), sometimes for several decades (Tables 8 and 9). However, leys of just 3 years generally had only small effects. Increases in SOC from growing leys compared to continuous arable crops is a genuine transfer of additional C from atmosphere to soil due to additional inputs of photosynthate entering the soil during the pasture phases of the rotation, mainly through the root mass. The issues when considering the wider adoption of this system mainly concern profitability at the farm scale. As discussed above in the context of manure use, many farmers in developed countries apparently find that the practical and economic benefits of specialization outweigh the less immediate benefits of improved soil quality that can be gained from a mixed animal/arable farming system. An expansion of mixed systems would require changes in policy and financial incentives and presupposes that there is a consumer demand for the animal products derived from the grass phase. Such a change would only be logical from consideration of climate change if it was accompanied by a decrease in the number of animals fed on grain in more intensive animal productions systems and probably an overall decrease in consumption of meat and dairy products (Bajželj et al., 2014). Such changes face major social and behavioural barriers and are unlikely to be achieved rapidly.

### 4.6 Addition of N fertilizer

In three examples from the Broadbalk Experiment, increasing the annual application of N fertilizer (with adequate supplies of other nutrients) caused increases in SOC >4‰ per year in the 0–23 cm soil layer that continued for periods of between 13 and 33 years (Table 6). This is consistent with earlier studies on Broadbalk (Glendining et al., 1996) and reviews of numerous experiments worldwide showing increased SOC in soils receiving N fertilizer compared to unfertilized or unbalanced fertilizer applications (Ladha, Reddy, Padre, & van Kessel, 2011; Han, Zhang, Wang, Sun, & Huang, 2016). It is presumed that this is due to increased inputs of organic C into soil from roots, exudates and above‐ground crop residues resulting from increased crop growth. It may also be that increased %N in these residues leads to greater conservation of organic C in soil organic matter. For regions of the world where fertilizer applications are currently low or nonexistent, this finding demonstrates a practical opportunity for increasing SOC with simultaneous benefits for crop production, provided other aspects of soil fertility, water availability and crop protection are in place. However, in such regions (e.g. Africa), there are major infrastructure and economic barriers to overcome to achieve rational fertilizer use. Where it does occur, it will be driven by food security goals, but improved soil quality will be a welcome co‐benefit. Increases in SOC derived in this way are unlikely to deliver climate change mitigation because of the greenhouse gas emissions associated with N fertilizer, especially direct and indirect N_2_O emissions, though yield‐scaled greenhouse gas emissions (i.e. emission per unit of agricultural product) may decrease (Linquist, van Groenigen, Adviento‐Borbe, Pittelkow, & van Kessel, 2012). In addition, there are large CO_2_ emissions associated with the manufacture of N fertilizer (Schlesinger, 2000), for example 3–8 t CO_2_‐equivalent per t N under European manufacturing conditions (Brentrup & Pallière, 2008). In regions where agriculture is well developed (e.g. Europe, North America), N fertilizer is already widely used, often at near‐optimum rates, so there is little opportunity to achieve increased SOC stocks through this mechanism. In regions where N is frequently overused (e.g. China) the overriding aim is to decrease applications to decrease water pollution and greenhouse gas emissions (Zhang et al., 2013): it is clearly not acceptable to justify excessively large N applications on the grounds of increasing SOC.

### 4.7 Practical limitations to achieving SOC increases of 4 per 1000 compared to initial SOC stock in agricultural soils

Any policies or initiatives, such as “4 per 1000”, that lead to increased SOC and remove CO_2_ from the atmosphere are to be welcomed. Even small increases in SOC are likely to improve soil quality and functioning for sustainable crop production, ecosystem services or both. But only in some cases will there be a concurrent benefit for climate change mitigation. Results from the long‐term experiments reviewed here demonstrate several land management practices that can increase SOC stocks, often at rates well above the 4‰ per year rate (in the 0–40 cm depth). However, as discussed above, there are many situations where these practices cannot be widely adopted, either because they are impractical for farmers or because of wider global issues. The limitations influencing widespread adoption vary between different regions of the world but mostly fall into one or more of the following categories:

1. Farmers do not have the necessary resources available, e.g. insufficient manure due to lack of animals or insufficient crop residues because they are required for other purposes, e.g. smallholder farmers in Africa (Thierfelder et al., 2013; Giller et al., 2015).
2. The practice is already widely used, giving limited scope for achieving increased SOC accumulation through greater adoption. An example is the return of crop residues to soil in many situations in Europe and North America.
3. Widespread adoption would have a negative impact on global food security, e.g. converting agricultural land to forest or grassland.
4. The necessary change of management would be uneconomic to the farmer under current conditions or impractical for some other reason. To implement such changes would probably require changes in government policies, regulations or subsidies to promote the practice.

For these reasons, we conclude that promoting the “4 per 1000” initiative as a major contribution to climate change mitigation is unrealistic. Of course, any contribution to mitigating climate change is beneficial but the reasoning we have set out, and that of Baveye et al. (2018), strongly indicate that the magnitude is far smaller than claimed, or implied, by the proposers of the initiative. We suggest that a more logical rationale for promoting practices that increase SOC is the urgent need to preserve and improve the functioning of soils, both for sustainable food security and wider ecosystem services. As discussed earlier, there is significant evidence that even small increases in SOC can have disproportionately large impacts on a range of soil physical properties so even very partial success in meeting the “4 per 1000” target can be highly beneficial in this respect. We recognize that this is a more nuanced argument with less obvious political appeal but it is more firmly based on sound evidence. It is also in line with attempting to meet the UN Sustainable Development Goals (http://www.un.org/sustainabledevelopment/sustainable-development-goals. We also emphasize that SOC increases are not necessarily the key issue when considering the success of climate change mitigation through land management practices: the impacts of management changes on emissions of trace greenhouse gases, especially N_2_O, must be given equal weight (Smith et al., 2008; Zhou et al., 2017; Tian et al., 2016).
