# Supplementary material for: Major limitations to achieving “4 per 1000” increases in soil organic carbon stock in temperate regions: Evidence from long‐term experiments at Rothamsted Research, United Kingdom
Source: Glob Chang Biol. 2018 Feb 28;24(6):2563–84. doi: 10.1111/gcb.14066 (PMC6001646; doi:10.1111/gcb.14066)

**Supporting Information:**

**Major limitations to achieving “4 per 1000” increases in soil organic carbon stock in temperate regions: evidence from long-term experiments at Rothamsted Research, UK**

**PAUL POULTON^1^, JOHNNY JOHNSTON^1^, ANDY MACDONALD^1^, RODGER WHITE^2^ and DAVID POWLSON^1^**

Experiment, site and number

(1) *Broadbalk Wheat, Rothamsted.* Available evidence suggests that the site had been in arable cropping for several centuries (Johnston & Garner, 1969; Rothamsted Research, 2006) before the experiment started in autumn 1843. Originally comprising large, long plots (mostly 300 x 6 m), it tests and compares inorganic fertilizers, including different amounts of fertilizer N, and farmyard manure (FYM), the latter on three plots which started in autumn 1843, in autumn 1884 or autumn 1967 (Rothamsted Research, 2006). Between 1926 and 1968 the experiment was divided into five sections and each section was bare fallowed, usually every fifth year, to control weeds by soil cultivation; FYM was not applied in these years. The five sections were halved in 1968 to create 10 sections; SOC data from three of the sections which reverted to continuous wheat have been used., On another section, straw has been incorporated since autumn 1986. On other sections within the experiment, and in other experiments, straw is removed unless it is applied as a treatment.

(2) *Hoosfield Barley, Rothamsted.* The site was also thought to have been in arable cropping for several centuries before the experiment started in 1852 (Jenkinson & Johnston, 1977). The initial large plots have been divided over the years; it tests and compares inorganic fertilizers and on two plots FYM applied since 1852 or 2001 (Rothamsted Research, 2006).

(3) & (4) *Fosters and Highfield Ley-arable experiments, Rothamsted*. These two experiments started in 1949 on sites with contrasting history (Johnston 1973; Johnston *et al*. 2009). On Fosters, which had been in long-term arable cropping, some plots stayed in continuous arable, some went into ley-arable rotations and some were sown to permanent grass. On Highfield, which had been in grass since 1838 (Lawes & Gilbert, 1885), some plots stayed in permanent grass, some went into continuous arable and some into ley-arable rotations.

(5) & 10) *Amounts of Straw experiments, Rothamsted and Woburn*. These fully replicated and randomized experiments started in 1986. The amount of straw added each autumn was based on that year’s straw yield and was applied at a zero, single, double and fourfold rate (Powlson *et al*., 2011).

(6) & (7) *Broadbalk and Geescroft Wildernesses, Rothamsted.* These two sites had previously been in long-term arable cropping before being abandoned in 1881 and 1886 respectively. Both sites now have naturally regenerated woodland (Poulton *et al*., 2003).

(8) *Park Grass, Rothamsted.* Started in 1856 on a site in pasture since *c.* 1700 (Lawes & Gilbert, 1859) this experiment tests effects of inorganic fertilizers and organic manures on the yields of permanent grass cut for hay in June and again in autumn. Treatments include FYM, applied once every four years since 1905, with either i) no additional treatment; ii) NPK in the other three years, or iii) fishmeal or poultry manure also applied once in four years (Warren & Johnston, 1964; Fornara *et al*, 2010).

(9) *Exhaustion Land.*  Started in 1852, this experiment on arable crops, tests the effects of fresh P and K fertilizers and of residues from fertilizers and FYM applied between 1856 and 1901 (Johnston & Poulton, 1977; Johnston *et al.,* 2017).

(11) *Organic Manuring, Woburn.* Since the experiment started in 1965, different organic amendments have been applied/or grown for limited periods; these have included FYM (at different rates), straw, commercially sourced compost, green manures and grass or grass/clover leys (Mattingly *et al*., 1974).

(12) *Green Manuring, Woburn.* From 1936-1967, the effects of different organic treatments were tested on various arable crops. Treatments included FYM, straw and green manures (Chater & Gasser, 1970).

(13) *Market Garden, Woburn.* Started in 1942, this experiment tested the effects of FYM, vegetable compost (replaced by FYM in 1962), sewage sludge and sludge compost (each applied at two rates) on the yields of market garden crops. Applications of sewage sludge and sludge compost stopped in 1962, and of FYM in 1968 (Johnston, 1975).

(14) *Ley-arable experiment, Woburn.* Started in 1938, the yields of crops grown in all-arable and ley-arable rotations are compared. FYM was applied every fifth year until the mid-1960s (Johnston *et al*., 2017).

(15) *Rotation II, Saxmundham.* Started in 1899, this experiment was designed to determine the best way of utilising limited amounts of manure and fertilizers in a 4-course rotation. FYM was applied once every four years (Mattingly *et al*., 1969).

(16) *Rotation I, Saxmundham.* This experiment also started in 1899 and featured a 4-course rotation. FYM applied every year was compared with inorganic fertilizers (Williams & Cooke, 1971).

Further information and data from some of the experiments, together with meteorological data is available through the Electronic Rothamsted Archive (e-RA; [www.era.rothamsted.ac.uk](http://www.era.rothamsted.ac.uk) ).

**Relating our data for increases in SOC with the 4 ‰ target**

The rates of increase in SOC calculated for our data are for soils taken to 20, 23 or 25 cm. Where possible, we have taken account of any extra soil that should have been sampled where bulk density declined (see Materials and Methods in main text). However, the “4 per 1000” initiative refers to increases in SOC to a depth of 40 cm. In several of the experiments reported here soil below 25 cm has been sampled and analysed so we can estimate how our measured increases to 25 cm relate to those for the 0-40 cm depth. For example, for the Woburn Organic Manuring experiment on a sandy loam soil (Mattingly *et al.,* 1974), in Table 2 the rate of increase in soil to 23 cm was 68.5 ‰, where FYM was given from 1965-1971, which equates to 40.7 ‰ when calculated for a 0-40 cm depth *i.e.* the rate of increase is *c.* 40% less for the larger amount of soil to 40 cm. Johnston *et al.* (2017) reported a similar difference for the Woburn Ley-arable experiment on the same soil type. On the silty clay loam at Rothamsted, we estimate for the Broadbalk experiment with only 1.0 – 1.3% SOC, the ‰ increase for the 0-40 cm depth is, on average, *c.*30-35% less than that calculated for the 0-23 cm depth. For example, for the period 1987-2000, after the amount of fertilizer N applied was increased from 48 to 192 kg ha^-1^, the rate of increase was 5.3 ‰ for the 0-23 cm depth (Table 6), which would equate to 3.8‰ for the soil to 40 cm. However, as the amount of carbon in the topsoil increases, forming a larger proportion of the total down to 40 cm, then the ‰ increase at 0-40 cm is only *c.* 20% less than that for the 0-23 cm depth. For example, the treatment receiving FYM since 1843 showed an annual increase of 11.2 ‰ from 1893-1914 for the 0-23 cm depth (Table S1) which equates to 9 ‰ for the soil to 40 cm. Based on these calculations, we suggest that, for the majority of the topsoil data presented here, an increase of 7 ‰ equates to 4 ‰ when expressed on a 0-40 cm basis. Where the topsoil contains a higher concentration of OC (*e.g.* in grassland or woodland sites or where large amounts of manure have been applied) an increase of *c*. 5 ‰ will equate to 4 ‰ for the 0-40 cm layer.

**References**

Chater M & Gasser JKR (1970) Effects of green manuring, farmyard manure, and straw on the organic matter of soil and of green manuring on available nitrogen. *Journal of Soil Science*, **21**, 127-137.

Fornara DA, Steinbeiss S, McNamara NP *et al.,* (2011) Increases in soil organic carbon sequestration can reduce the global warming potential of long-term liming to permanent grassland. *Global Change Biology,* **17**, 1925-1934. doi:10.1111/j.1365-2486.2010.02328.x

Jenkinson DS & Johnston AE (1977) Soil Organic Matter in the Hoosfield Continuous Barley Experiment. Rothamsted Experimental Station, Report for 1976, Part 2, 87-101. Lawes Agricultural Trust, Harpenden, UK. https://doi.org/10.23637/ERADOC-1-34448

Johnston AE (1973) The Effects of Ley and Arable Cropping Systems on the Amounts of Soil Organic Matter in the Rothamsted and Woburn Ley-Arable Experiments. Rothamsted Experimental Station, Report for 1972, Part 2, 131-159. Lawes Agricultural Trust, Harpenden, UK. <https://doi.org/10.23637/ERADOC-1-34692>

Johnston AE (1975) The Woburn Market Garden Experiment, 1942-69: II. The Effects of the Treatments on Soil pH, Soil Carbon, Nitrogen, Phosphorus and Potassium. Rothamsted Experimental Station, Report for 1974, Part 2, 102-131. Lawes Agricultural Trust, Harpenden, UK. https://doi.org/10.23637/ERADOC-1-33162

Johnston AE & Garner HV (1969) The Broadbalk Wheat experiment: historical introduction. Rothamsted Experimental Station, Report for 1968, Part 2, 12-25. Lawes Agricultural Trust, Harpenden, UK. https://doi.org/10.23637/ERADOC-1-34916

Johnston AE & Poulton PR (1977) Yields on the Exhaustion Land and Changes in the NPK Content of the Soils due to Cropping and Manuring, 1852-1975. Rothamsted Experimental Station, Report for 1976, Part 2, 53-85. Lawes Agricultural Trust, Harpenden, UK. https://doi.org/10.23637/ERADOC-1-34447

Johnston AE, Poulton PR & Coleman K (2009) Soil Organic Matter: It’s Importance in Sustainable Agriculture and Carbon Dioxide Fluxes. *Advances in Agronomy.***101,** 1-57

Johnston AE, Poulton PR, Coleman K, Macdonald AJ & White RP (2017) Changes in soil organic matter over 70 years in continuous arable and ley-arable rotations on a sandy loam soil in England. *European Journal of Soil Science,* **68**, 305-316. doi: 101111/ejss.12415

Lawes JB & Gilbert JH (1885) On some points in the composition of soils; with results illustrating the sources of the fertility of Manitoba prairie soils. *Journal of the Chemical Society,* **XLVII**, 380-422.

Mattingly GEGM, Johnston AE & Chater M (1969) The Residual Value of Farmyard Manure and Superphosphate in the Saxmundham Rotation II Experiment, 1899-1968. Rothamsted Experimental Station, Report for 1969, Part 2, 91-112. Lawes Agricultural Trust, Harpenden, UK. <https://doi.org/10.23637/ERADOC-1-34865>

Mattingly GEGM, Chater M & Poulton PR (1974) The Woburn Organic Manuring Experiment: II. Soil Analyses, 1964-72, with Special Reference to Changes in Carbon and Nitrogen. Rothamsted Experimental Station, Report for 1973, Part 2, 134-151. Lawes Agricultural Trust, Harpenden, UK. https://doi.org/10.23637/ERADOC-1-34636

Poulton PR, Pye E, Hargreaves PR & Jenkinson DS (2003) Accumulation of carbon and nitrogen by old arable land reverting to woodland. *Global Change Biology,* **9**, 942-955.

Powlson DS, Glendining MJ, Coleman K & Whitmore AP (2011) Implications for soil properties of removing cereal straw: results from long-term studies. *Agronomy Journal,* **103**,279–287. doi:10.2134/agronj2010.0146s

Rothamsted Research (2006) Guide to the Classical and other Long-term Experiments, Datasets and Sample Archive. Lawes Agricultural Trust, Harpenden, UK. <https://doi.org/10.23637/ROTHAMSTED-LONG-TERM-EXPERIMENTS-GUIDE-2006>

Tinsley J (1950) The determination of organic carbon in soils by dichromic mixtures. In: *Transactions of the 4^th^ International Congress of Soil Science,* (eds; FA van Baren, J Doeksen, WR Domingo, JChL Favejee, HJ Hardon, EG Mulder, PK Peerlkamp, AC Schuffelen, JG Vermaat, WC Visser, AJ Zuur & JJ Schuurman), **1**, pp. 161–164), Hoitsema Brothers, Amsterdam.

Warren RG & Johnston AE (1964) The Park Grass Experiment. Rothamsted Experimental Station, Report for 1963, 240-262. Lawes Agricultural Trust, Harpenden, UK. https://doi.org/10.23637/ERADOC-1-38770

Williams RJB & Cooke GW (1971) Results of the Rotation I Experiment at Saxmundham, 1964-69. Rothamsted Experimental Station, Report for 1970, Part 2, 68-97. Lawes Agricultural Trust, Harpenden, UK. https://doi.org/10.23637/ERADOC-1-34801

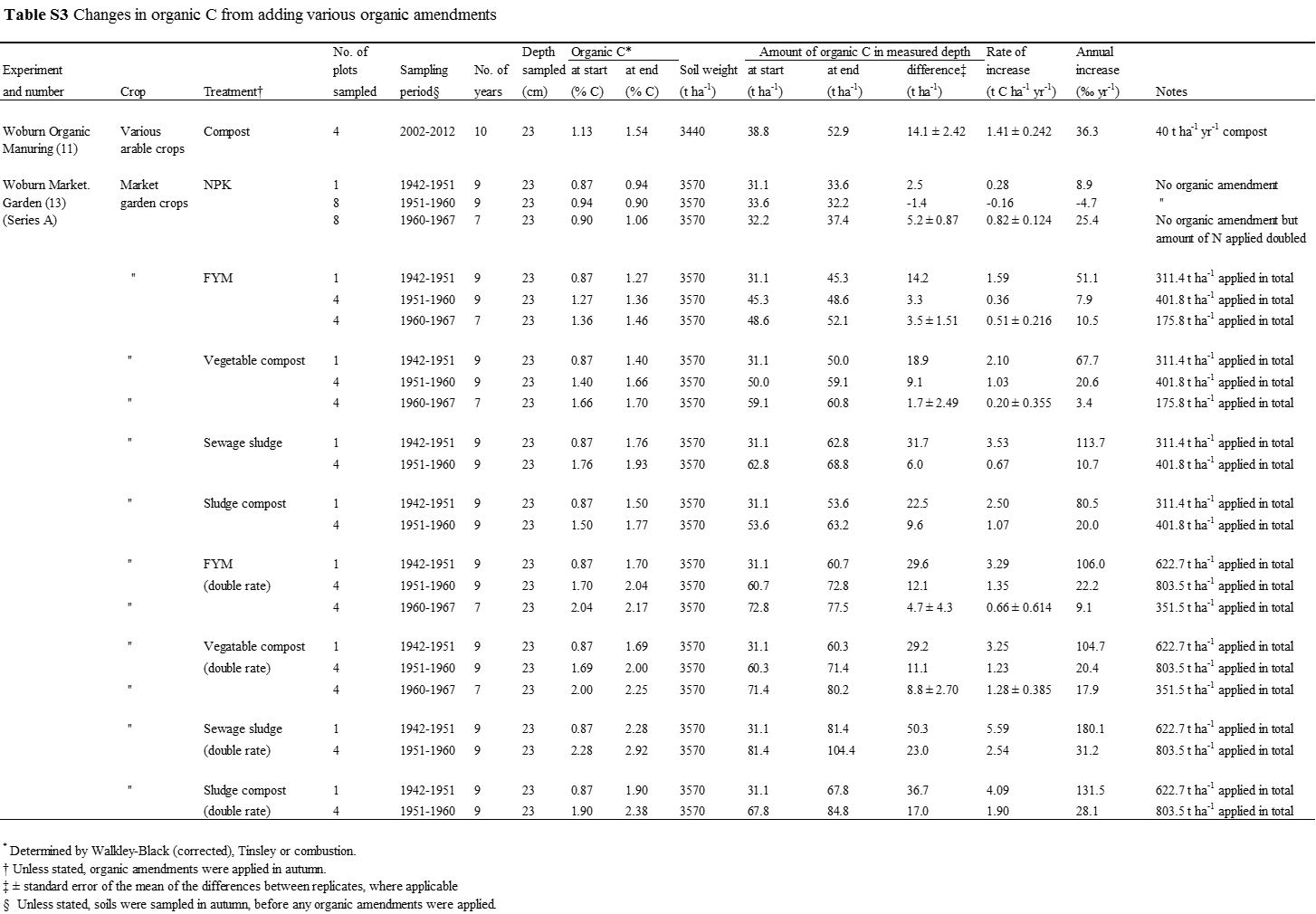


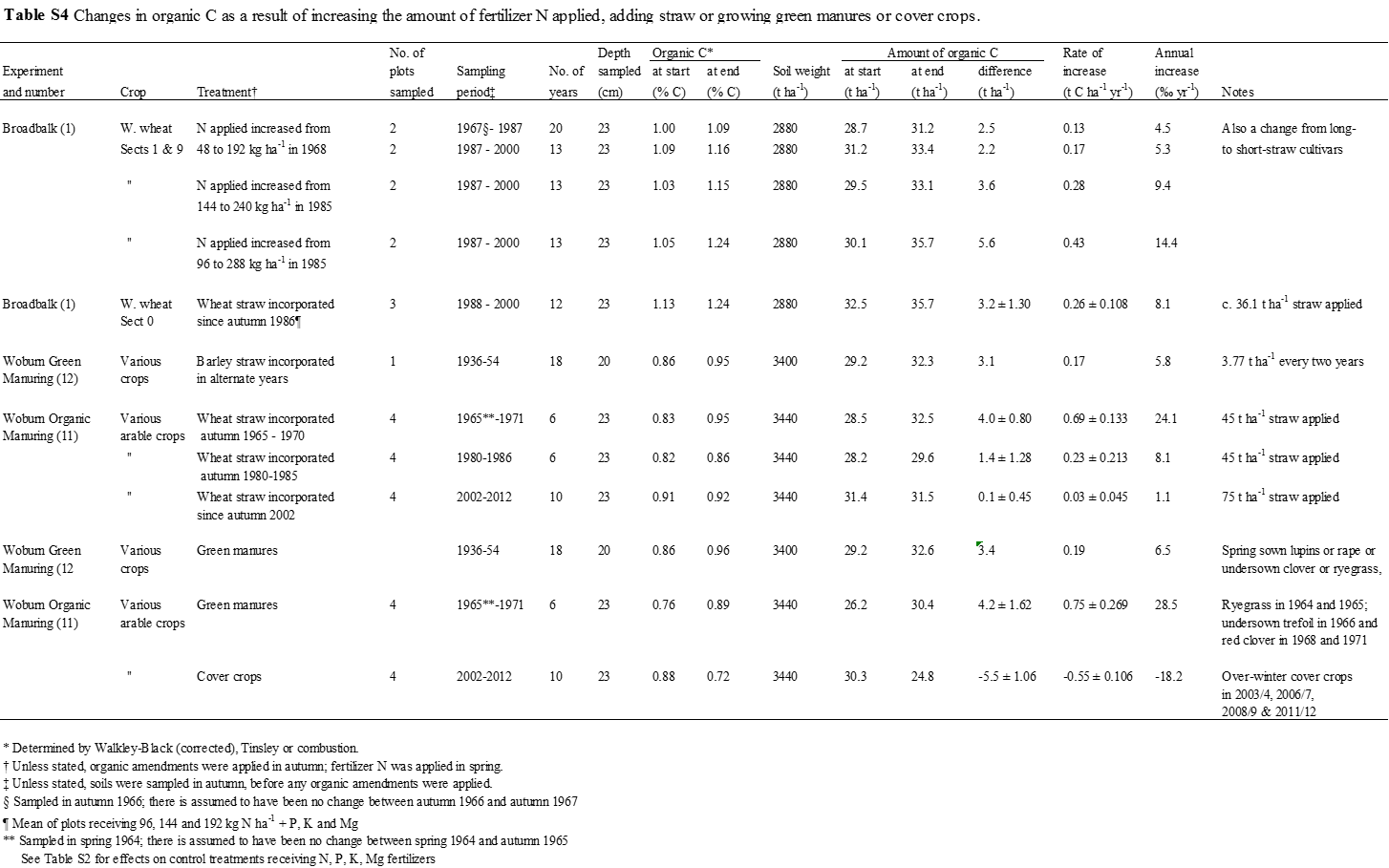


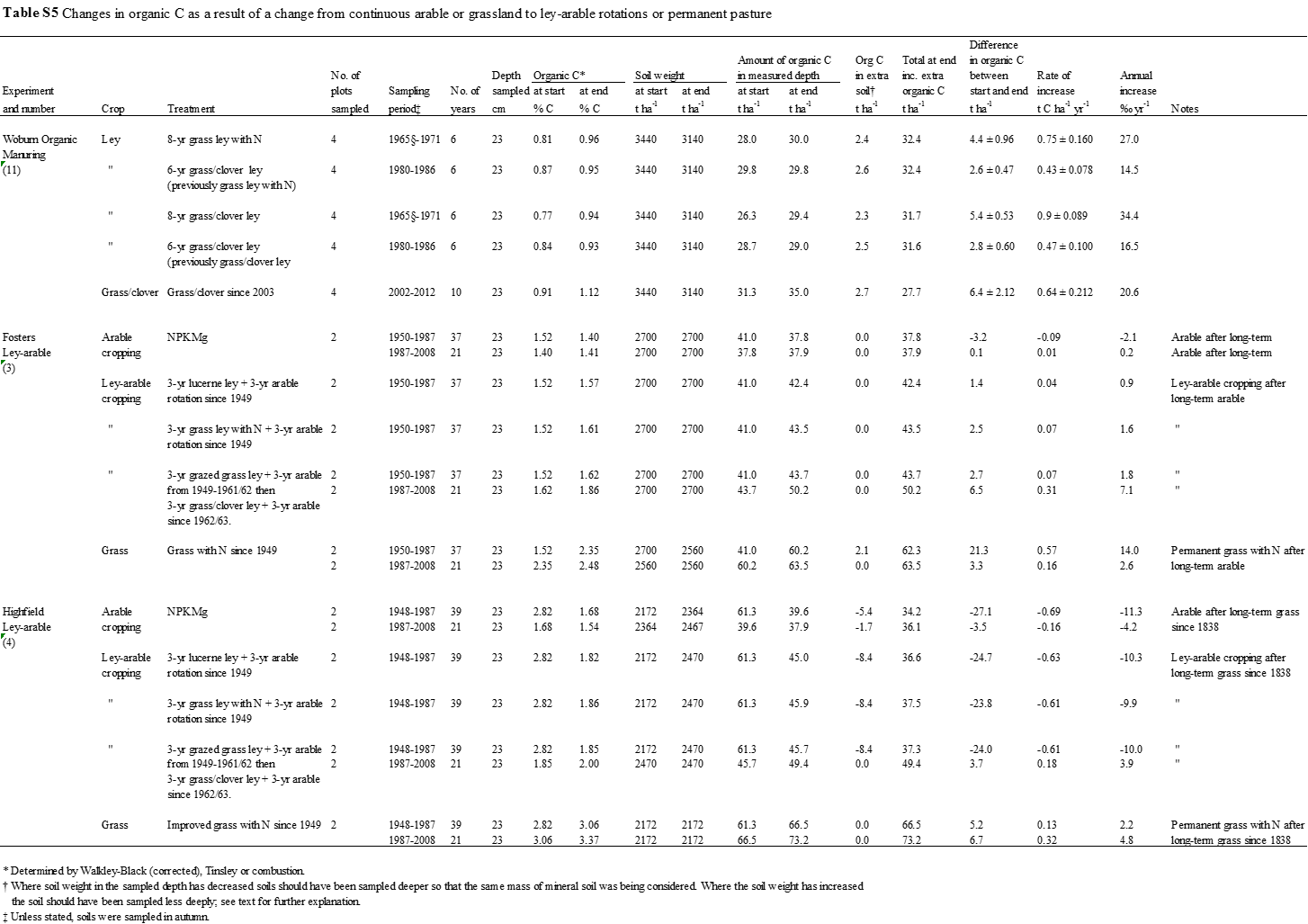

Supplement: Supplementary file 1 [file GCB-24-2563-s001.docx]
